# Extracellular-Vesicle-Associated Nucleic Acids in the Diagnosis and Treatment of Respiratory Diseases: A Narrative Review

**DOI:** 10.3390/pharmaceutics18080945

**Published:** 2026-07-30

**Authors:** Shuairong Lin, Ruixu Lan, Xiaoyan Zhu, Rui Shen, Ruiying Liu, Jinzhou Cheng, Xiaoliu Liu

**Affiliations:** School of Medicine, Wuhan University of Science and Technology, Wuhan 430065, China

**Keywords:** extracellular vesicles, nucleic acids, respiratory diseases, biomarkers, RNA therapy

## Abstract

Respiratory diseases impose a substantial global burden; however, early diagnosis, disease-activity monitoring, and the clinical translation of nucleic acid therapeutics are constrained by the lack of robust biomarkers and efficient delivery systems. This narrative review focuses on four classes of RNA—messenger RNA (mRNA), circular RNA (circRNA), small interfering RNA (siRNA), and microRNA (miRNA)—using exosomes as a representative subtype of extracellular vesicles (EVs) to discuss EV biogenesis, transport, uptake, and engineered cargo loading. We summarize the diagnostic and therapeutic applications of EV-associated nucleic acids in chronic or non-severe respiratory diseases, including asthma, chronic obstructive pulmonary disease, idiopathic pulmonary fibrosis, and cystic fibrosis, as well as in severe acute conditions such as acute respiratory distress syndrome and severe pneumonia. Biofluid-derived EV-associated RNAs can reflect inflammation, immune dysregulation, epithelial injury, infection, and fibrosis, supporting their potential use in disease classification, monitoring, and prognostic assessment. Natural EVs may modulate inflammation and tissue repair through their endogenous cargo, while engineered EVs can deliver therapeutic nucleic acids to exert anti-inflammatory, anti-infective, antifibrotic, and barrier-restorative effects. However, clinical translation is limited by non-standardized isolation and characterization methods, product heterogeneity, variable cargo loading, and insufficient stability and quality-control frameworks. Continued advances in EV isolation, characterization, nucleic acid loading, potency assessment, and manufacturing control are required to realize the diagnostic and therapeutic potential of EV-associated nucleic acids in respiratory diseases.

## 1. Introduction

Respiratory diseases are one of the leading causes of global deaths and disability, creating a long-term, heavy public health and socioeconomic burden. Global burden studies indicate that respiratory diseases affect a large population and cause substantial mortality. In 2023, there were about 569 million patients with chronic respiratory diseases and about 4.2 million deaths worldwide [1]. Lower-respiratory-tract infections accounted for approximately 2.5 million deaths and 98.7 million disability-adjusted life years (DALYs) [2]. Together, these findings highlight the substantial effects of respiratory diseases on mortality, quality of life, and healthcare-resource utilization, underscoring their importance as a global public health priority. The diagnosis and clinical assessment of respiratory diseases primarily depend on symptoms, pulmonary function testing, and imaging [3]. However, these methods have limited capacity to detect early changes in disease activity or to predict acute exacerbations. Current pharmacological and supportive treatments can alleviate symptoms, control infections, and reduce inflammation [4,5], but they often do not fully prevent disease progression. Consequently, further research is essential to identify molecular markers indicative of early pulmonary inflammation and injury as well as to develop therapies aimed at regulating aberrant gene expression and disease-associated pathways.

Nucleic acid molecules provide new opportunities for biomarker discovery and therapeutic intervention in respiratory diseases. Disease-associated RNA alterations may serve as candidate biomarkers, whereas engineered mRNAs, circRNAs, siRNAs, and miRNAs can be used for protein expression or gene regulation [6,7,8,9,10].

However, there are still bottlenecks in the clinical application of nucleic acid molecules. Naked RNA or oligonucleotides are easily degraded by nucleases, and it is difficult for them to cross the cell membrane into the target cell by themselves [11]. Their clinical application therefore requires delivery systems that protect the cargo and facilitate uptake by target cells. Extracellular vesicles (EVs) are lipid bilayer membrane particles released by cells into the extracellular environment. They can carry various biomolecules, such as proteins, lipids, and RNA, and participate in intercellular information transmission [12,13]. As naturally derived membrane vesicles, EVs are being investigated as candidate carriers for therapeutic nucleic acids [14]. In disease or inflammatory states, the endogenous nucleic acid cargo in EVs from body fluids has potential biomarker value [15]. At the same time, natural EVs themselves can participate in immunomodulation and tissue repair through endogenous nucleic acids and other cargo [16], and engineered EVs can also load therapeutic nucleic acids and participate in the treatment of diseases [17].

Existing reviews [15,18,19] mostly start from the overall function of EVs or a single nucleic acid cargo, but few have employed EV-associated nucleic acids as the main thread to systematically organize and discuss body-fluid-derived EV nucleic acid markers, the role of endogenous nucleic acids in natural EVs, and engineered EV nucleic acid delivery in the diagnosis and treatment of respiratory diseases. This review examines EV-associated nucleic acids as potential biomarkers and therapeutic agents in respiratory diseases. We focus on chronic and non-severe conditions, such as asthma, chronic obstructive pulmonary disease (COPD), idiopathic pulmonary fibrosis (IPF), and cystic fibrosis (CF), as well as severe acute conditions, including acute respiratory distress syndrome (ARDS) and severe pneumonia. We also discuss the biological basis, engineering strategies, methodological limitations, and translational challenges associated with EV-based nucleic acid applications.

## 2. Biological Characteristics and EV-Related Applications of Four RNA Classes in Respiratory Diseases

**Figure 1:**
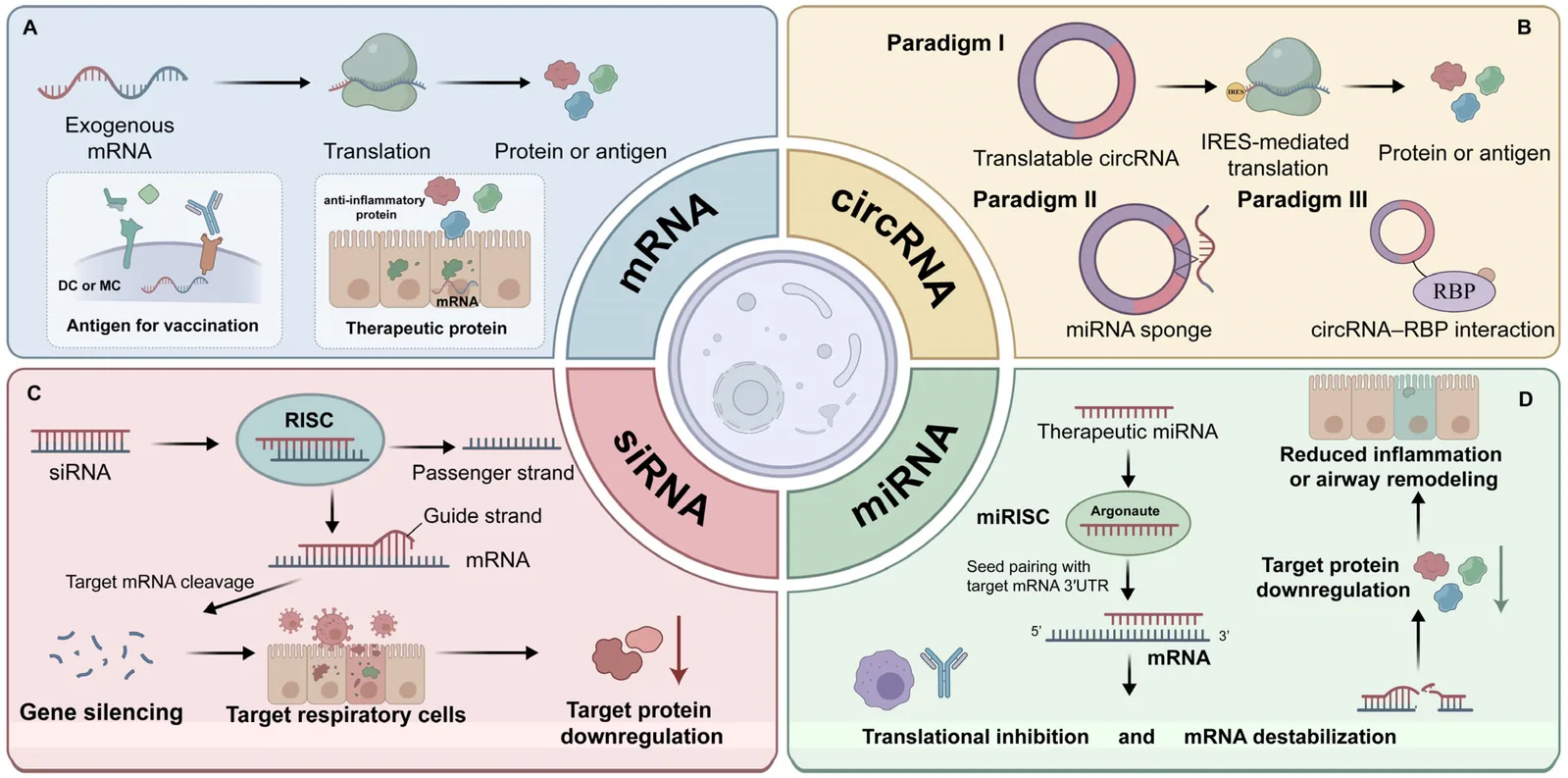
Principal mechanisms of EV-associated RNAs in respiratory disease therapy. (**A**) The delivered mRNA is translated into therapeutic proteins or antigens within the cytoplasm. (**B**) Circular RNA (circRNA) may facilitate cap-independent translation, act as a microRNA (miRNA) sponge, or interact with RNA-binding proteins (RBPs). (**C**) Small interfering RNA (siRNA) directs the RNA-induced silencing complex (RISC) to cleave highly complementary target mRNA. (**D**) The miRNA-containing miRISC represses translation or promotes the degradation of partially complementary target mRNAs. Abbreviations: circRNA, circular RNA; EV, extracellular vesicle; IRES, internal ribosome entry site; miRNA, microRNA; miRISC, miRNA-induced silencing complex; mRNA, messenger RNA; RBP, RNA-binding protein; RISC, RNA-induced silencing complex; siRNA, small interfering RNA; UTR, untranslated region.

This review focuses on four RNA classes relevant to respiratory disease biomarkers or therapeutics: messenger RNA (mRNA), circular RNA (circRNA), small interfering RNA (siRNA), and microRNA (miRNA) (Figure 1). These RNA classes differ in their structures, mechanisms of action, diagnostic relevance, and therapeutic applications. The following subsections describe their principal characteristics and associations with EVs, as summarized in Table 1.

### 2.1. mRNA

mRNA is a single-stranded RNA molecule that conveys genetic information from DNA to the translational machinery, serving as a template for protein synthesis [20]. Cellular mRNA profiles can provide information about cell identity and functional state [21,22]. However, compared to miRNA and circRNA, the application of mRNA in the diagnosis of respiratory diseases is still relatively limited. This may be related to factors such as long mRNA molecules and susceptibility to degradation [23].

Therefore, in respiratory diseases, mRNA is more often used as a nucleic acid therapeutic molecule that encodes therapeutic proteins or antigens [24], while disease diagnosis mainly focuses on non-coding RNAs such as miRNA and circRNA. After exogenous mRNA enters the target cytoplasm, it can directly use the translation system of the host cell to synthesize the target protein and exert the therapeutic function of the target protein [24,25]. Unlike DNA-based therapeutics, mRNA does not require nuclear entry and carries no inherent risk of genomic integration. However, its safety remains contingent upon factors such as sequence design, formulation, dosage, and route of administration [24,26]. mRNA also offers substantial coding capacity and design flexibility, allowing therapeutic constructs to be tailored to specific disease targets [24,27]. Based on this mechanism, mRNA therapy can be used not only to express exogenous antigens to induce specific immune responses but also to express missing therapeutic proteins to achieve protein replacement therapy. EVs have been reported to contain either full-length or fragmented mRNAs. In certain experimental settings, they have also been shown to transfer translatable mRNA to recipient cells [28,29,30]. This provides a theoretical basis for EV mRNA as a disease state marker and for the delivery of therapeutic mRNA.

### 2.2. circRNA

circRNAs are a class of covalently closed single-stranded RNA primarily generated through the back-splicing of precursor mRNA [31]. The lack of free 5′ and 3′ ends grants them greater resistance to exonuclease-mediated degradation compared to linear RNA. Some naturally occurring or engineered circRNAs can also facilitate cap-independent translation driven by internal ribosome entry sites or N6-methyladenosine modifications, allowing for relatively sustained protein expression [32,33]. CircRNAs have been reported to be enriched in EV preparations, where vesicular encapsulation may offer additional protection against extracellular degradation [34,35]. Consequently, EV-associated circRNAs have been explored as diagnostic biomarkers and potential therapeutic platforms.

The biological effects of circRNAs are not mediated through a single pathway but depend on their subcellular localization and interacting partners. In the nucleus, some intron-containing circRNAs can interact with transcription complexes such as U1 snRNP and RNA polymerase II, thus enhancing the transcription output of the parent gene at the cis level [36,37]. In the cytoplasm, circRNAs containing miRNA-binding sites can sequester specific miRNAs through a competing endogenous RNA (ceRNA) mechanism. This interaction reduces miRNA-mediated repression of target mRNAs, thereby altering downstream gene expression [38,39]. In addition, circRNA can also participate in post-transcriptional regulation through direct interaction with RNA-binding proteins (RBPs). RBPs are widely involved in the processing, nuclear export, stability maintenance, and regulation of translation efficiency of mRNA. RBP-mediated RNA regulation can therefore modulate the activity of multiple signaling pathways involved in inflammation and fibrosis [40]. In this context, circRNA can competitively bind to specific RBPs to change their interaction with mRNA or their subcellular distribution, thus altering the stability and translation of mRNA, and ultimately affecting the direction and intensity of gene expression related to inflammation or fibrosis [41].

Certain circRNAs may mitigate airway inflammation and remodeling by modulating miRNA- or RBP-dependent pathways and promoting the expression of proteins involved in anti-inflammatory, antifibrotic, and tissue-repair processes [39,42]. Engineered circRNAs can support the prolonged expression of therapeutic proteins or antigens, potentially offering a longer duration of translation compared to comparable linear mRNAs [32].

### 2.3. siRNA

siRNAs are typically 21–23-nucleotide RNA duplexes. The RNA interference (RNAi) mechanism was first discovered in a gene silencing experiment triggered by double-stranded RNA in Caenorhabditis elegans (nematodes) [43], and then Elbashir et al. confirmed that about 21 nt siRNA duplexes can achieve sequence-specific gene silencing in mammalian cells [44]. Therapeutic siRNAs are typically synthetic duplexes engineered to silence specific genes, including those associated with viral infections and disease-related host genes. These molecules are primarily utilized as interventional agents rather than as endogenous diagnostic biomarkers.

After siRNA enters the cytoplasm, it is loaded into the RISC. The complex retains the guide strand, which directs RISC to the complementary target mRNA. Within RISC, Ago2 mediates target-mRNA cleavage, thereby promoting mRNA degradation and suppressing protein expression [45]. Unlike mRNA and circRNA, which produce therapeutic proteins through the cellular translation system, siRNA can downregulate the expression of pathogenic genes and inhibit the production of pathogenic proteins, so it is more suitable for diseases such as overactivation of signaling axes or viral replication [46]. It is worth noting that unlike the multi-target regulation of miRNA, siRNA usually acts against a single target mRNA, which typically has higher specificity and silencing efficiency for a single specific target gene [47] and can be used to inhibit respiratory virus replication or downregulate single pathogenic proteins. In addition, because siRNA is easily degraded by nucleases in the body and is difficult to enter target cells, EVs are currently regarded as ideal delivery carriers for siRNA [48,49]. Engineered EV-loaded siRNA has achieved target gene silencing and inflammation relief in animal models of respiratory diseases [50].

### 2.4. miRNA

miRNA is a small endogenous non-coding RNA, and the length of mature miRNA is about 20–23 nt [51,52]. miRNA research can be traced back to the discovery of lin-4 in Caenorhabditis elegans. lin-4 was shown to repress LIN-14 protein expression by binding to complementary sequences in the 3′ untranslated region (3′ UTR) of lin-14 mRNA [51]. This finding suggested that small RNAs can participate in post-transcriptional gene regulation. In animal cells, miRNA usually does not need to be fully complementary to the target mRNA, but mainly relies on its 5′-terminal seed sequence to identify the binding site in the target mRNA 3′-UTR, and mediates translation inhibition, mRNA deadenylation, and degradation through the RNA-induced silencing complex [53]. Therefore, a single miRNA can often regulate multiple target mRNAs at the same time, and multiple miRNAs can also work together on the same mRNA, showing obvious network characteristics [52].

miRNA is one of the most studied small RNAs in EV-associated nucleic acid research. EVs are present in a variety of biofluids, and their lipid bilayer can protect intravesicular miRNAs from degradation by extracellular nucleases [54]. Extracellular vesicle (EV) cargo is influenced by cell type and the local microenvironment. Consequently, disease-associated alterations in EV-miRNA profiles may indicate inflammatory activation, immune dysregulation, oxidative stress, and epithelial injury [55]. Therefore, body-fluid-derived EV-miRNA has the potential to be a liquid biopsy marker for respiratory diseases.

In addition, miRNA can be used as an intervention molecule to regulate abnormal gene expression and pathological pathways. Unlike siRNA, which mainly silences a single target mRNA through highly complementary pairing, miRNA usually regulates multiple target genes at the same time through partial complementarity [56]. Although miRNAs may offer reduced target specificity compared to siRNAs, their capacity to modulate interconnected gene networks presents potential advantages in the context of multifactorial diseases, such as chronic airway inflammation and COPD [57,58]. In current studies, downregulated protective miRNAs can be restored using miRNA mimics or agomirs, whereas abnormally upregulated pathogenic miRNAs can be inhibited using antagomirs, anti-miR oligonucleotides, or miRNA sponge/decoy strategies [59,60].

**Table 1:** Comparison of the biological characteristics and applications of mRNA, miRNA, circRNA, and siRNA.

RNA Category	Biological Function	Stability	Diagnostic Applications	Therapeutic Applications	Major Delivery Challenges
mRNA	Serves as a template for the synthesis of encoded proteins or antigens.	The unprotected form of mRNA is relatively unstable. As a long, linear molecule, mRNA is prone to degradation; however, encapsulation within EVs can safeguard it and enhance its delivery to recipient cells.	The diagnostic applications of EV-associated mRNA remain relatively limited.	Can be used for protein replacement and vaccination [24].	Its high molecular weight, structural complexity, and susceptibility to nuclease degradation make efficient loading and intracellular delivery technically challenging.
circRNA	circRNA is a class of covalently closed RNA molecules that can regulate the transcription of their parental genes, act as miRNA sponges, interact with RBPs, and facilitate cap-independent translation.	Due to the lack of free 5′ and 3′ ends, circRNAs exhibit a high resistance to exonuclease-mediated degradation, thus maintaining relative stability when enriched in EVs.	EV-associated circRNA has demonstrated potential as a stable biomarker for both the diagnosis and monitoring of disease progression. Research has explored these applications in conditions such as IPF and ARDS [61,62].	circRNA can facilitate the sustained expression of therapeutic proteins or antigens while also regulating inflammatory, fibrotic, and tissue repair pathways through mechanisms involving miRNA sponging and RBPs [32,39,42].	The mechanisms underlying the sorting of circRNAs into EVs remain incompletely understood. Further investigation is required for efficient loading, purification of EVs, quality control, and the establishment of standardized delivery strategies.
siRNA	Through highly complementary base pairing, siRNA directs Ago2 within the RISC to identify and cleave the target mRNA, thereby facilitating sequence-specific gene silencing.	Naked siRNA is rapidly degraded by nucleases in vivo; however, encapsulation in EVs can protect the siRNA and enhance its delivery to target cells.	siRNA is primarily an artificially designed tool for gene silencing and is generally not utilized as an endogenous biomarker for diseases.	siRNA can mediate highly specific silencing of viral genes or other disease-causing genes [46].	siRNA is susceptible to degradation by nucleases and exhibits limited ability to enter target cells. The efficiency of its loading into EVs, along with its cellular uptake and tissue- and cell-specific targeting, necessitates further optimization.
miRNA	As an endogenous post-transcriptional regulator, miRNA binds to partially complementary sequences in target mRNAs, mediating translational repression or mRNA degradation. This mechanism allows miRNAs to simultaneously regulate multiple target genes and associated pathways.	The lipid bilayer of EVs serves to protect encapsulated miRNAs from degradation by extracellular nucleases, thereby enhancing their stability in biological fluids.	EV-associated miRNA can reflect inflammatory activation, immune dysregulation, oxidative stress, and epithelial injury [55].	Protective miRNAs can be restored using miRNA mimics, while pathogenic miRNAs can be inhibited via anti-miRs and related strategies. This modulation affects aberrant gene expression and multiple pathological pathways [59,60].	Naked miRNA is readily degraded by nucleases and does not efficiently cross cell membranes. Its multi-target regulatory characteristics, EV loading, dose standardization, and targeted delivery necessitate further optimization.

Abbreviations: Ago2, Argonaute 2; anti-miR, anti-microRNA oligonucleotide; ARDS, acute respiratory distress syndrome; circRNA, circular RNA; EV, extracellular vesicle; IPF, idiopathic pulmonary fibrosis; miRNA, microRNA; mRNA, messenger RNA; RBP, RNA-binding protein; RISC, RNA-induced silencing complex; siRNA, small interfering RNA.

## 3. EV Classification, Exosome Biogenesis, and the Biological Basis of Nucleic Acid Delivery

EVs can be categorized into three main types based on their biogenesis: exosomes, which originate from the endosomal system; microvesicles, which are formed by outward budding from the plasma membrane; and apoptotic bodies, which are released during the process of apoptosis [12].

Apoptotic bodies are usually larger than 1000 nm and are formed in the late stage of cell apoptosis. Their contents are mainly organelle fragments and nucleic acid components [63,64]. Microvesicles, which are mostly 100–1000 nm in diameter, are formed by outward budding directly from the plasma membrane [63,65]. Unlike apoptotic bodies and microvesicles, exosomes are small extracellular vesicles with a diameter of about 30–150 nm. Exosomes are generated through the endosomal pathway. They are released as intraluminal vesicles (ILVs) when multivesicular bodies (MVBs) fuse with the plasma membrane [66]. CD9, CD63, CD81, and other marker molecules are common on their membrane surface, and bioactive components such as nucleic acids and proteins can be carried inside [28,67]. Due to the extensive study of their endosomal biogenesis, as well as the ability to engineer their membranes and cargo, exosomes are frequently investigated in research related to nucleic acid biomarkers and delivery mechanisms [68]. Therefore, after introducing the overall classification of EVs, this review focuses on the biogenesis, release, in vivo transport, and uptake by recipient cells of exosomes, and introduces the biological basis for detecting body fluid EV nucleic acids and delivering nucleic acid drugs.

Because EV subtypes cannot be assigned solely on the basis of particle size or a limited set of molecular markers, the terminology used in this review follows the level of biogenetic evidence reported in the original studies, as further discussed in Section 3.5 [12]. The disease-specific sections maintain the terminology utilized in the original studies. Preparations identified as exosomes are referred to as such only when substantiated by the reported biogenetic evidence; otherwise, the original terms ‘EVs’ or ‘small EVs’ are preserved.

### 3.1. Exosome Biogenesis and Secretion

The classical biogenesis of exosomes begins with endocytosis to form the early endosome. Within early endosomes, cargo undergoes initial sorting. Some of it is recycled to the plasma membrane or returned to the Golgi apparatus, while the other part is retained in the late endosome as the endosome matures [69]. As the endosome matures, its limiting membrane invaginates into the lumen and pinches off, forming multiple intraluminal vesicles (ILVs). At this time, the late endosome rich in ILVs is called a multivesicular body (MVB) [70]. Therefore, the exosome is not formed by outward budding from the plasma membrane; it is the product of the gradual maturation and sorting of the endosomal system.

ILV formation and RNA cargo sorting jointly contribute to exosome biogenesis. ILVs can be formed through both ESCRT-dependent and ESCRT-independent mechanisms. In the canonical ESCRT pathway, ESCRT complexes sequentially participate in cargo clustering and endosomal membrane remodeling, while ESCRT-III and VPS4 are primarily involved in bud-neck constriction and membrane scission [71,72]. The selective sorting of RNA cargo is influenced by various factors, including RNA sequence and structural features, RBPs, the endosomal sorting machinery, and the physiological or activation state of donor cells [71,72,73,74]. Under particular cellular conditions, certain RNA–RBP complexes can be recruited to multivesicular bodies and sites of ILV formation through interactions with ESCRT-associated factors such as HRS [71,74]. In addition to this canonical mechanism, ESCRT-independent pathways also contribute to ILV formation. A common mechanism involves nSMase2-mediated ceramide generation, which promotes inward budding by altering membrane lipid composition and inducing negative membrane curvature [71,72,75]. However, these two types of mechanisms are not entirely distinct; different cells and cargoes may utilize partially overlapping sorting and vesicle-budding programs.

miRNA is one of the most studied categories of exosomal nucleic acid cargo. The sorting of certain miRNAs into exosomes is facilitated by RBPs, such as hnRNPA2B1, SYNCRIP, and YBX1. These proteins recognize specific sequence motifs and promote the incorporation of miRNAs into the ILVs during exosome biogenesis [76,77,78,79]. After recognition, the corresponding miRNA is more likely to be incorporated into the forming ILVs and finally released with the exosome. Therefore, the composition of miRNAs in exosomes is not only affected by the expression level of miRNAs in donor cells but also regulated by the sorting mechanism mediated by RNA-binding proteins.

The mechanisms governing the sorting of longer RNAs remain poorly defined. Exosomal mRNA fragments are often enriched in 3′ UTR sequences, indicating that specific cis-acting elements may play a role in vesicular loading [80,81,82]. In contrast, while circRNAs can be enriched in EV preparations, the sequence, structural, and RBP-dependent mechanisms responsible for their sorting are still not fully characterized [34].

**Figure 2:**
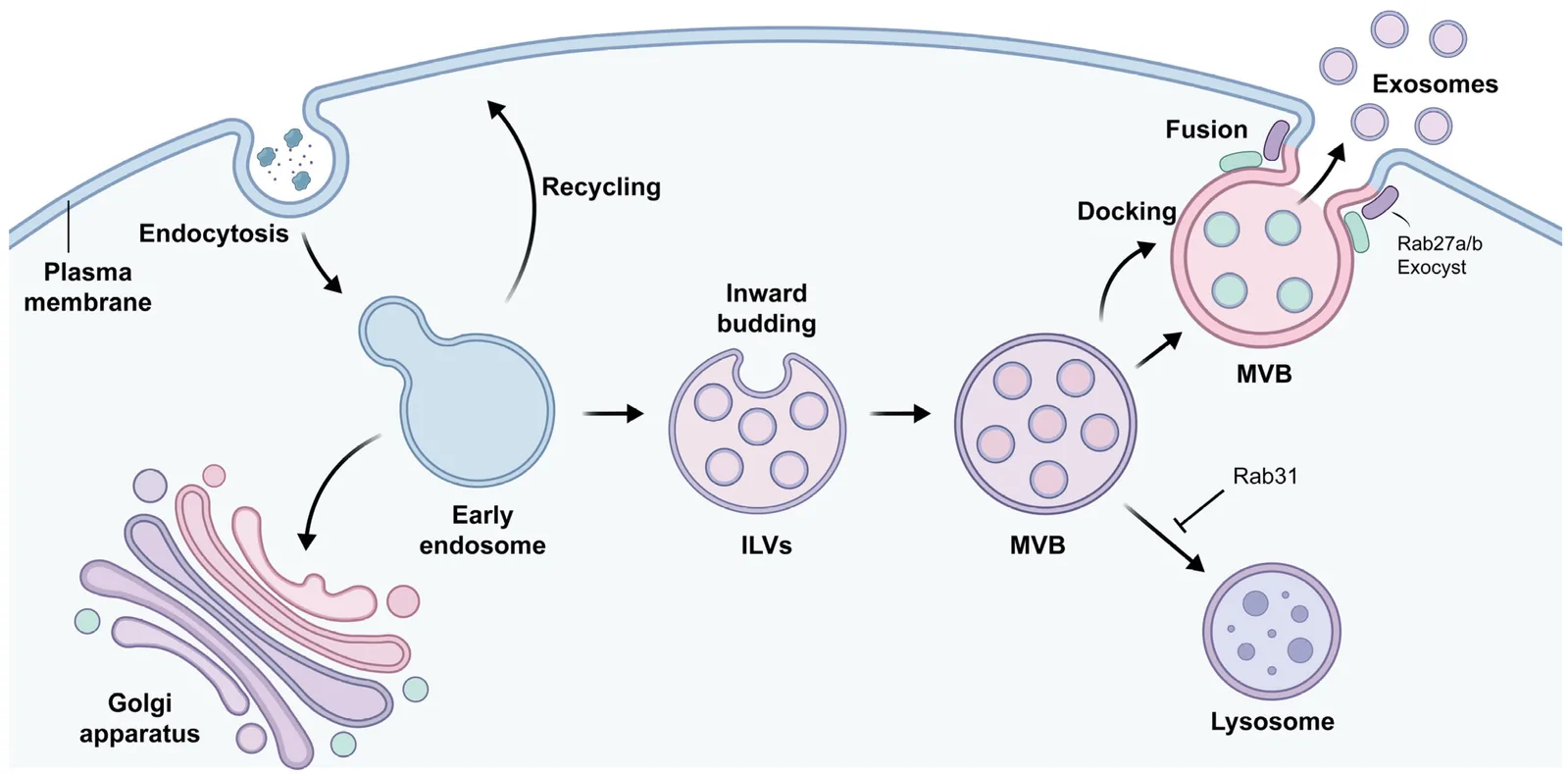
Biogenesis and Release Process of Exosomes. The classical biogenesis of exosomes begins with endocytosis to form early endosomes. Upon entering early endosomes, the cargo undergoes initial sorting; some components are recycled to the plasma membrane or returned to the Golgi apparatus, while others are retained in late endosomes as the endosomes mature. As these endosomes progress in maturation, their limiting membrane continuously invaginates into the lumen and undergoes scission, resulting in the generation of multiple intraluminal vesicles (ILVs). At this stage, late endosomes enriched with ILVs are referred to as multivesicular bodies (MVBs). MVBs can follow two primary pathways: one involves fusion with lysosomes, leading to the degradation of their contents; the other entails transport to the cell periphery, where, regulated by Rab27a/b and the exocyst, they dock and fuse with the plasma membrane, ultimately releasing the ILVs contained within the MVB into the extracellular space to form exosomes. Furthermore, Rab31 can inhibit the transport of MVBs to the lysosomal degradation pathway, thereby promoting a regulatory mechanism that facilitates the release of ILVs in the form of exosomes. Abbreviations: EVs, extracellular vesicles; ILVs, intraluminal vesicles; MVB, multivesicular body; exocyst, exocyst complex.

Following their formation, MVBs undergo one of two principal fates: lysosomal degradation or secretion. If the MVB fuses with lysosomes, its internal ILVs and the proteins, lipids, and nucleic acids contained within them will be degraded in the acidic hydrolytic environment [83]. If the MVB is directed to the secretory pathway, it must first be transported to the cell periphery and undergo tethering, docking, and fusion with the plasma membrane under the synergistic action of Rab family small GTPases and the exocyst complex. The ILVs are then released into the extracellular space as exosomes [84]. Ostrowski et al. [85] found that Rab27a and Rab27b are involved in positioning secretory MVBs near the plasma membrane. At the same time, the exocyst complex acts as a tethering complex that stabilizes the docking state by connecting the MVB to the plasma membrane and creating conditions for membrane fusion [84]. In addition, the fate of MVBs (degradation vs. secretion) is jointly regulated by membrane lipid composition, endosomal acidification, lysosomal function, and the Rab7-related transport network [83]. For example, Rab31 can inhibit Rab7 activity by recruiting TBC1 domain family member 2B (TBC1D2B), thus reducing MVB transport to the lysosomal degradation pathway, and when endosome-lysosome fusion is blocked or lysosomal function is impaired, exosome release increases [83,86]. The specific formation pathway is shown in Figure 2.

### 3.2. Exosome Transport and Uptake by Recipient Cells

After their release, exosomes may be taken up by neighboring cells or transported through the tissue interstitium, influenced by interactions with the extracellular matrix (ECM), interstitial fluid flow, and barriers posed by vascular or lymphatic systems [87,88]. In local tissues, exosomes mainly migrate over short distances. Their movement is not simply dependent on passive diffusion but is also affected by the flow of interstitial fluid and interactions with the ECM [87]. Exosomes can not only diffuse through the interstitial space but also bind to matrix components such as collagen, laminin, fibronectin, and glycosaminoglycans (GAGs), and then be taken up by neighboring cells [89]. Therefore, the ECM is not only the physical environment for exosome movement but also affects their diffusion range to a certain extent.

When exosomes mediate long-distance cell communication, they can be further transported through the lymphatic system and blood circulation [90,91]. The lymphatic pathway is an important route through which exosomes can enter draining lymph nodes from peripheral tissues. Studies have shown that exosomes can reach draining lymph nodes via lymphatic flow and can subsequently be taken up by macrophages, B cells, and lymphatic endothelial cells, thus affecting antigen presentation and immune cell function status and participating in innate immune activation, adaptive immune response activation, and immune tolerance regulation [92,93]. After entering the blood circulation, exosomes can participate in remote organ communication, but whether they move from tissues into the circulation or from the circulation into distal tissues, they must cross an endothelial barrier [90]. In pathological conditions such as inflammation, increased endothelial permeability further promotes the exchange of exosomes between tissues and circulation [94]. In the lungs, exosome transport also has local microenvironmental characteristics, which can be affected by factors such as the airway mucus layer, mucociliary clearance, alveolar macrophage uptake, and changes in alveolar-capillary barrier permeability [95,96].

To exert their biological effects after local or systemic distribution, exosomes must be retained within target tissues, interact with recipient-cell surfaces, and undergo cellular uptake or membrane fusion. Some exosomes carry integrins on their surface. Integrins are transmembrane adhesion receptors composed of α and β subunits, which can affect the adhesion of exosomes to the local matrix or recipient cell membrane [97]. Classic studies have shown [98] that different integrin combinations in tumor-derived exosomes are associated with organ tropism, among which α6β4 and α6β1 are linked to lung metastasis, and αvβ5 to liver metastasis. Blocking α6β4 or αvβ5 reduces exosome uptake and metastasis formation in the corresponding tissues. In addition, tetraspanins such as CD9, CD63, and CD81 are involved in the organization of membrane microdomains and the interaction between exosomes and recipient cells [99]. CD47 can weaken clearance by the mononuclear phagocyte system (MPS) by binding to signal regulatory protein α (SIRPα) on the surface of phagocytes, thereby prolonging the retention time of exosomes in circulation [100,101].

**Figure 3:**
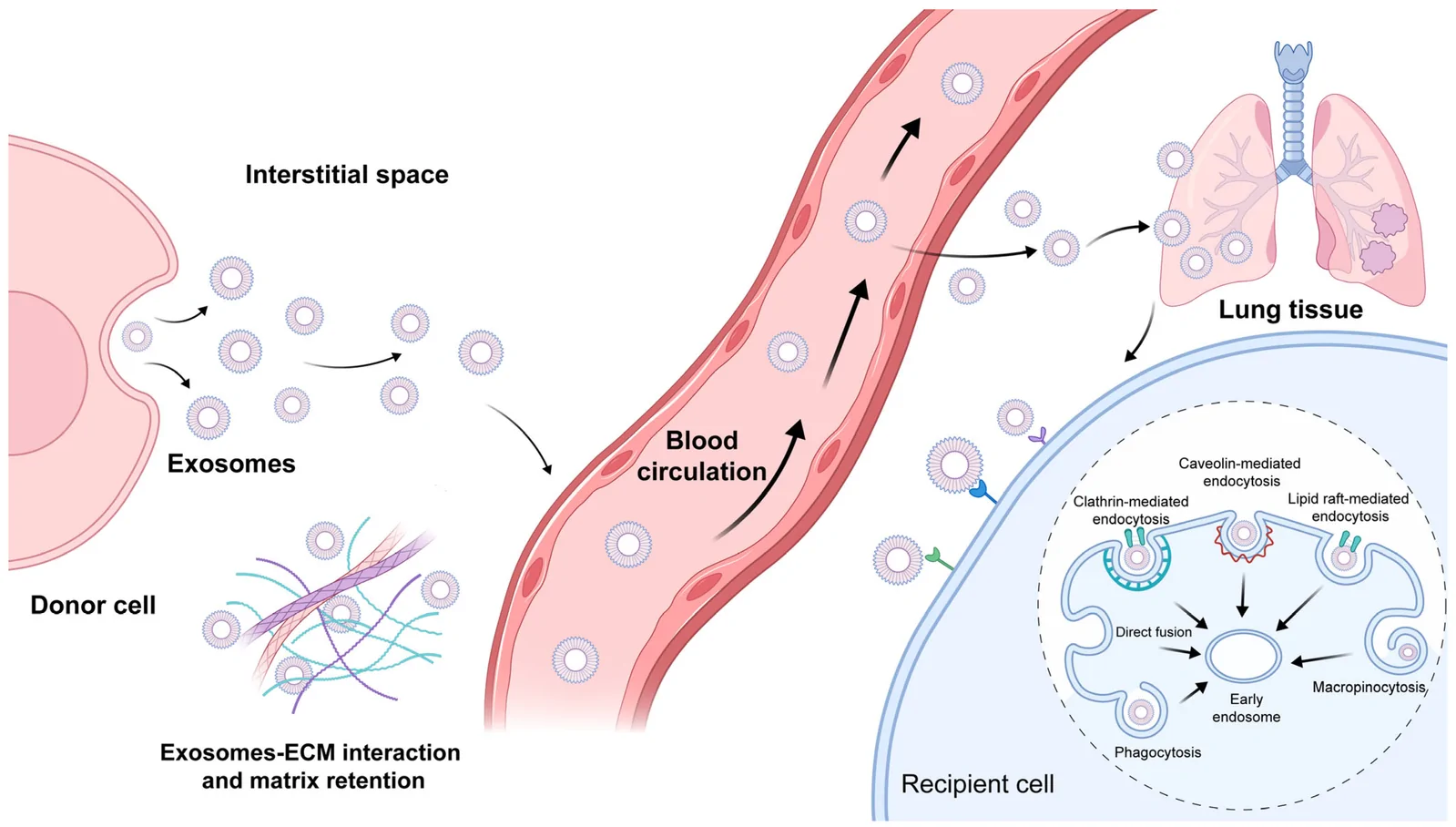
Schematic diagram of exosome transport in the extracellular microenvironment, tissue distribution, and recipient-cell uptake mechanisms. Exosomes secreted by donor cells initially enter the intercellular space, where a portion interacts with ECM, resulting in local retention and participation in the regulation of the local tissue microenvironment. The remaining exosomes continue to diffuse and subsequently enter the bloodstream, where they are transported via the circulatory system to distant tissues. In applications related to respiratory system diseases, either circulating exosomes or locally released exosomes can reach lung tissue and interact with receptors, adhesion molecules, or membrane lipid structures present on the surface of target cells in the lungs. At the site of recipient cells, exosomes can be internalized or deliver their contents through various mechanisms. The figure illustrates six common uptake pathways, including direct fusion of the exosomal membrane with the recipient cell plasma membrane, clathrin-mediated endocytosis, caveolin-mediated endocytosis, lipid raft-mediated endocytosis, macropinocytosis, and phagocytosis. Abbreviations: ECM, extracellular matrix.

After the initial adhesion between exosomes and recipient cells, exosomes can further enter cells via endocytosis, phagocytosis, or membrane fusion, but the selectivity and efficiency of cellular entry are influenced by factors such as surface ligands on exosomes and membrane molecules on recipient cells [98,102,103]. Subsequently, exosomes enter recipient cells. Currently, six common entry mechanisms have been described: clathrin-mediated endocytosis, caveolin-mediated endocytosis, lipid raft-mediated endocytosis, macropinocytosis, phagocytosis, and direct membrane fusion (Figure 3), among which endocytosis is the main pathway for exosome entry into cells [104,105,106]. Clathrin-mediated endocytosis and caveolin-related endocytosis are classic receptor-dependent internalization processes [106]. It is worth noting that EVs from the same source can simultaneously enter different cells via multiple uptake pathways [99].

### 3.3. Biological Heterogeneity of EV Populations

EV populations display significant biological heterogeneity. Variations in cellular origin, biogenesis pathways, and donor-cell states contribute to the formation of distinct EV subpopulations, each characterized by unique surface molecules, nucleic acid cargoes, and functional properties. Furthermore, even among EVs released by a single cell type, the molecular composition of individual EVs is not entirely uniform [12,71,107]. In diagnostic studies, these variations may yield insights into cellular origin and disease state. However, EV nucleic acid profiles in biofluids typically consist of mixed signals from multiple cellular sources and EV subpopulations, with low-abundance disease-associated subpopulations potentially diluted in bulk analyses, thereby complicating biomarker discovery and validation efforts [107,108]. In therapeutic contexts, the variations in cargo content and biological potency among EV subpopulations further complicate the evaluation of product potency and the maintenance of batch-to-batch consistency [14,107].

### 3.4. EV Sample Processing, Separation, and Enrichment for Nucleic Acid Analysis

The separation and enrichment of EVs from biofluids or cell-culture supernatants are essential prerequisites for subsequent nucleic acid analysis. Due to their close resemblance to non-vesicular particles, such as lipoproteins, protein aggregates, and ribonucleoprotein complexes, in terms of size and density, no single separation method can currently achieve an optimal balance among recovery, specificity, and vesicle integrity. The MISEV2023 guidelines recommend selecting separation strategies based on the sample type, research objectives, and downstream analytical requirements. When non-EV components cannot be adequately excluded, the resulting samples should preferably be referred to as an ‘EV preparation’ or ‘EV-containing preparation’ [12]. Furthermore, all studies should comprehensively report the sample source, initial volume, processing time, procedures used to remove cells and debris, storage conditions, and the number of freeze–thaw cycles [12].

Common methods for EV isolation include differential ultracentrifugation, size-exclusion chromatography, polymer-based precipitation, immunoaffinity capture, and microfluidic technologies. Differential ultracentrifugation is effective for processing relatively large sample volumes; however, it may co-isolate protein aggregates and lipoproteins. Size-exclusion chromatography operates under relatively mild conditions but fails to completely eliminate non-EV particles of similar size, potentially leading to sample dilution. Polymer-based precipitation is straightforward and offers relatively high recovery rates, yet it can inadvertently enrich proteins, lipoproteins, and non-vesicular RNA. Immunoaffinity capture allows for the enrichment of EV subpopulations that express specific surface markers, but it does not provide a comprehensive representation of the entire EV population. Microfluidic technologies require only small sample volumes and enable rapid processing; however, methodological standards remain inconsistent across platforms [12,109]. When high specificity is essential, combining separation methods based on different principles may be required, although this typically results in reduced EV recovery.

Methods for EV separation and RNA extraction can significantly influence the proportions of EV subpopulations and co-isolated non-vesicular components, which in turn affects RNA yield, species composition, and relative abundance. Consequently, discrepancies in findings related to EV-miRNA, EV-circRNA, or EV-mRNA across different studies may stem not only from biological variations but also from differences in sample pre-processing, EV separation, and RNA extraction methodologies [110,111].

Before conducting RNA profiling, it is essential to establish the relationship between the detected RNA and EVs. Merely detecting RNA in an EV-enriched preparation does not confirm that the RNA resides within the vesicles, as RNA may also be associated with the EV surface or originate from co-isolated lipoproteins and ribonucleoprotein complexes. Furthermore, total RNA content cannot serve as a substitute for evaluating EV concentration or purity [12,112,113].

Currently, the selection of EV RNA sequencing methods should be guided by the specific target RNA species. Small RNA sequencing is primarily employed for the detection of miRNAs and other short RNAs, while low-input total RNA sequencing is suitable for analyzing mRNAs, circRNAs, and various non-coding RNAs. In data analysis, circRNAs are typically identified based on reads that span back-splice junctions, in conjunction with appropriate bioinformatics tools [110,114]. Due to the low abundance of EV RNA, technical biases may be introduced during RNA extraction, adapter ligation, reverse transcription, and PCR amplification. Furthermore, short-read sequencing is inadequate for determining mRNA integrity based solely on local sequence reads, and different circRNA detection tools may yield inconsistent results. Therefore, studies should comprehensively report RNA extraction, library preparation, sequencing, and analytical procedures, and validate significant candidate RNAs using independent methods such as RT-qPCR [110,114].

### 3.5. EV Characterization, Nomenclature, and Reporting

Following EV separation or enrichment, the resulting preparations should be characterized using methods based on complementary principles. Since no single technique can simultaneously assess particle number, structure, molecular composition, and non-EV components, it is essential to combine particle concentration and size analysis, morphological examination, and molecular marker detection [12]. Nanoparticle tracking analysis can estimate particle concentration and size distribution; however, it cannot distinguish EVs from lipoproteins and protein aggregates. Electron microscopy can visualize membranous vesicular structures but is limited in determining their specific biogenesis pathways. Therefore, studies should report the complete particle size distribution, instrument and software parameters, and methodological detection limitations, as well as the sample preparation, image acquisition, and analytical conditions employed in electron microscopy [12].

MISEV2023 employs a five-category protein framework to evaluate EV preparations. Category 1 includes transmembrane or lipid-anchored proteins associated with the plasma membrane or endosomal membrane, such as CD9, CD63, and CD81. Category 2 encompasses EV-associated cytosolic proteins, including TSG101, ALIX, and syntenin. Category 3 consists of common non-EV co-isolated components, such as apolipoproteins, albumin, and immunoglobulins. Categories 4 and 5 are utilized to provide supplementary information regarding intracellular origin, secreted proteins, or EV surface-corona components [12]. In practice, appropriate markers should be selected from Categories 1 and 2, and corresponding non-EV components should be evaluated based on the sample source.

In instances where the biogenesis pathway of EVs has not been directly demonstrated, it is preferable to use the term ‘EV’ or an operational term based on criteria such as size, density, cellular origin, or molecular phenotype, such as ‘small EVs’ or ‘CD63-positive EVs’ [12]. A preparation cannot be classified as exosomes solely based on a particle size range of 30–150 nm or positivity for markers such as CD9, CD63, and CD81. When summarizing previous studies, the terminology used in the original publications may be preserved; however, the results should be interpreted with caution, taking into account the extent of EV separation and characterization.

## 4. Foundation for the Application of EV-Associated Nucleic Acids in the Diagnosis and Treatment of Respiratory Diseases

**Figure 4:**
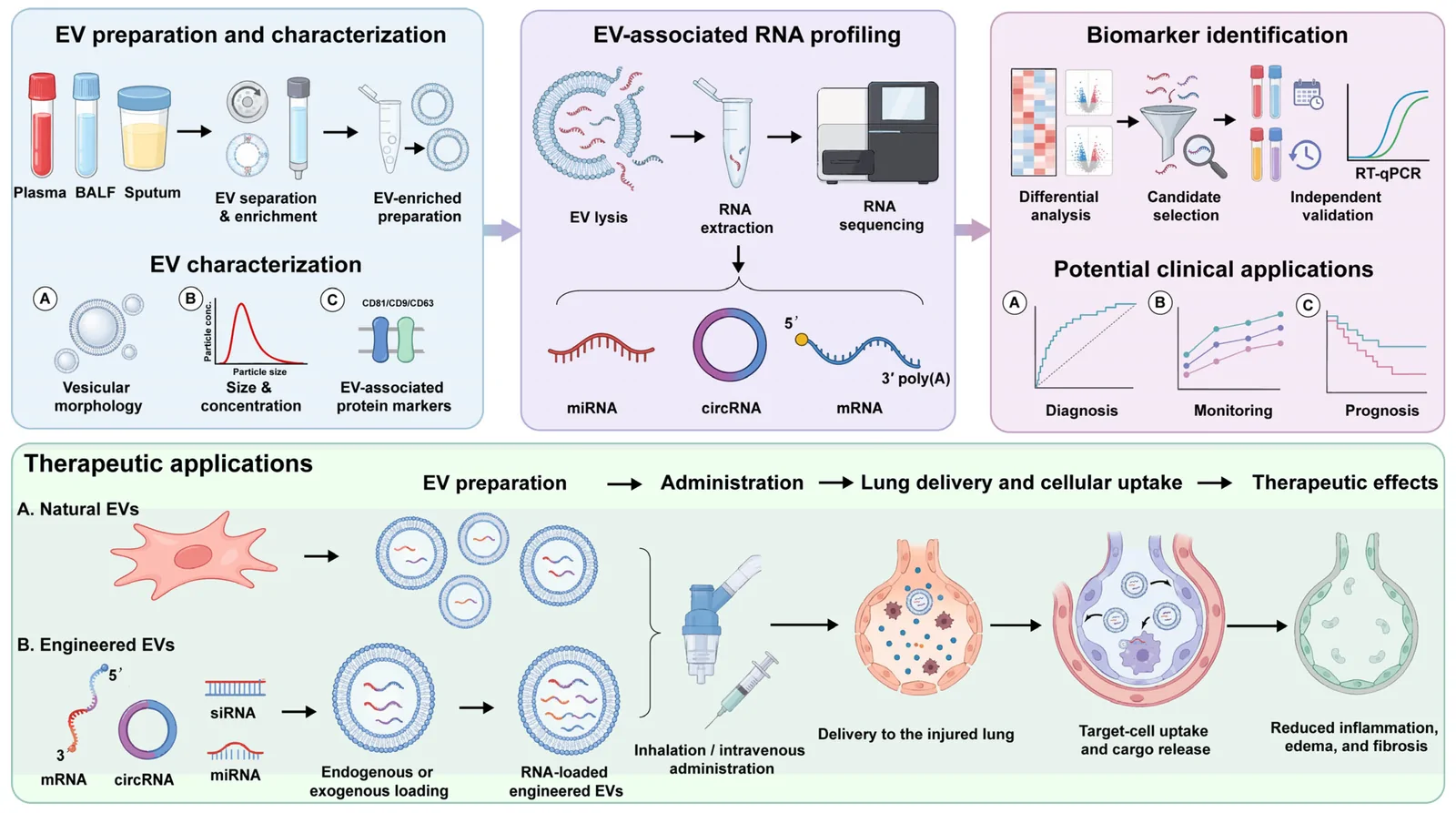
Integrated workflow for the diagnostic and therapeutic application of EV-associated nucleic acids in respiratory diseases. Biofluids such as plasma, bronchoalveolar lavage fluid (BALF), and sputum undergo processes including extracellular vesicle (EV) separation, characterization, RNA extraction, and transcriptomic profiling. Candidate EV-associated RNAs are identified and subsequently validated in independent cohorts using reverse transcription quantitative polymerase chain reaction (RT-qPCR) for their potential application in diagnosis, monitoring, or prognostic assessment. For therapeutic purposes, natural EVs or engineered EVs loaded with mRNA, circular RNA (circRNA), small interfering RNA (siRNA), or microRNA (miRNA) may be administered either intravenously or via inhalation. Following uptake by target lung cells and subsequent functional cargo release, these EVs may mitigate inflammation, edema, and fibrosis while promoting barrier repair. Abbreviations: BALF, bronchoalveolar lavage fluid; CD, cluster of differentiation (CD9, CD63, and CD81); circRNA, circular RNA; EV, extracellular vesicle; miRNA, microRNA; mRNA, messenger RNA; poly(A), polyadenylate tail; RNA, ribonucleic acid; RT-qPCR, reverse transcription quantitative polymerase chain reaction; siRNA, small interfering RNA.

The applications of EV-associated nucleic acids in respiratory diseases can be categorized into two primary paradigms. The first paradigm focuses on profiling nucleic acid cargo in biofluid-derived EVs for the purposes of disease classification, monitoring, and prognostic assessment. The second paradigm involves the use of either natural or engineered EVs to deliver functional nucleic acids that can modulate inflammation, infection, fibrosis, or barrier injury. Building on the mechanisms of EV biogenesis, release, and uptake described above, we summarize the integrated workflow from EV preparation and RNA profiling to biomarker identification and therapeutic application in respiratory diseases in Figure 4.

Diagnostic studies frequently analyze EVs isolated from blood, bronchoalveolar lavage fluid (BALF), sputum, and various other biofluids. Compared with direct detection of free nucleic acids, the EV lipid bilayer membrane can protect intravesicular RNA from extracellular ribonuclease (RNase) degradation to a certain extent, making it more suitable as a stable detection target in liquid biopsy [54,95]. Because the nucleic acids, proteins, and other cargo carried by EVs are affected by donor cell type, inflammatory stimulation, infection status, and the tissue damage microenvironment, the body fluid EV nucleic acid profile can reflect, to a certain extent, inflammatory activation, abnormal immune cell response, epithelial damage, oxidative stress, fibrosis, and other disease processes in respiratory diseases [15,95,115]. In addition, different body fluid samples reflect different information. Blood-derived EVs are easier to obtain and are suitable for reflecting systemic inflammation and disease activity [95]. BALF- and sputum-derived EVs are closer to the local lung microenvironment and are more likely to reflect inflammation, infection, and barrier damage in the airways or alveoli [95].

As discussed above, therapeutic nucleic acids generally require delivery vehicles to improve their stability and cellular uptake [116]. In respiratory diseases, pulmonary delivery presents additional barriers, including airway mucus, mucociliary clearance, alveolar macrophage uptake, and the alveolar epithelial and vascular endothelial barriers [117]. An effective pulmonary nucleic acid delivery system should therefore protect its cargo while promoting deposition and retention at the target site and uptake by relevant lung cells.

Lipid nanoparticles (LNPs) are currently among the most clinically advanced platforms for nucleic acid delivery [116]. A typical LNP is composed of ionizable lipids, cholesterol, phospholipids, and polyethylene glycol (PEG)-modified lipids. This formulation can efficiently encapsulate siRNA, mRNA, and other nucleic acid molecules and has been validated in mRNA vaccines and several nucleic acid drugs [116,118,119]. Its advantages include high transfection efficiency and effective intracellular delivery [120]. Nevertheless, LNP performance varies substantially across tissues and cell types. Especially after intravenous administration, traditional LNPs tend to accumulate in the liver [121] and pose certain safety concerns (such as immunogenicity and toxicity) [122]. To apply LNPs to lung diseases, further improvement in delivery efficiency to the lungs and specific cells is needed through lipid composition optimization, surface targeting modifications, or local administration [123].

Compared with LNPs, EVs are membranous vesicles released by cells with a natural lipid bilayer structure [12]. The RNA enclosed in the vesicular lumen is protected by the membrane structure, reducing its risk of degradation by extracellular RNases [12,28]. As described in Section 3.2, native EV membrane molecules can significantly influence tissue interactions and cellular uptake. However, these endogenous characteristics do not guarantee efficient or cell-specific delivery; thus, additional engineering may be necessary to enhance delivery efficacy.

EVs and LNPs each have distinct advantages and limitations. LNPs possess a relatively well-defined lipid composition, and the standardization of nucleic acid encapsulation, dose control, and large-scale manufacturing is more readily achievable; additionally, their clinical translation is more advanced. However, many systemically administered LNPs exhibit liver tropism, and lipid or PEG components may elicit immune responses, while endosomal escape remains a significant barrier to cytosolic delivery [116,124,125]. Conversely, EVs feature a natural membrane architecture and cell-derived molecules, exhibiting favorable biocompatibility in some preparations; however, challenges persist in controlling their nucleic acid loading capacity, cytosolic release, product heterogeneity, and batch-to-batch consistency [14].

EV-related therapeutic applications can be further divided into natural EV treatment and engineered EV delivery of therapeutic nucleic acids. The therapeutic effects of natural EVs arise from the combined activities of endogenous nucleic acids, proteins, lipids, and other cargoes released by donor cells. For example, mesenchymal-stem-cell-derived EVs (MSC-EVs) can exert immunomodulatory, anti-inflammatory, anti-oxidative stress, and epithelial/endothelial barrier repair effects through their own miRNA, protein, and lipid molecules [126,127]. Engineered EV development generally involves two distinct components: nucleic acid loading and surface modification for targeted delivery.

Currently, the methods for nucleic acid loading into engineered EVs primarily include endogenous and exogenous loading [128]. Endogenous loading involves the transfection, viral transduction, or genetic engineering of donor cells, which facilitates the incorporation of target nucleic acids into vesicles during EV biogenesis [129,130]. This method presents the advantage of minimizing direct disruption to the EV membrane while allowing for the concurrent expression of nucleic acid-sorting elements or targeting membrane proteins. However, the engineering of donor cells can be relatively complex, and the expression level of RNA does not necessarily correlate with the amount loaded into EVs.

Certain engineering platforms have demonstrated the potential to significantly enhance both EV production and nucleic acid loading. For instance, an improved endogenous loading platform utilizing Transwell-based asymmetric cell electroporation achieved a more than 30-fold increase in EV release compared to untreated cells. When calculated as the total number of RNA molecules divided by the number of EV particles, the population-averaged loading levels at peak production were 8.2 ± 1.5 copies of TP53 mRNA per EV and 1899.9 ± 114.5 copies of siKRAS^G12D^ per EV [131].

Exogenous loading involves the introduction of nucleic acids into EVs after their isolation through various methods, including incubation, electroporation, sonication, freeze–thaw cycling, or extrusion [132,133,134]. This approach offers greater flexibility, allowing for the independent control of the input amounts of EVs and RNA. However, it may lead to issues such as RNA aggregation or adsorption to the outer membrane, damage to the EV membrane, vesicle aggregation, and significant nucleic acid loss. A comparative study on mRNA loading indicated that some methods could associate approximately 70% of EVs with mRNA, with the freeze–thaw method demonstrating the highest overall performance [135]. A classical study on siRNA electroporation revealed that, after accounting for false-positive signals resulting from RNA precipitation, siRNA retention was below 0.05%. This suggests that RNA signals detected in EV pellets may overestimate the actual loading efficiency [132]. Therefore, quantitative comparisons among different strategies should concurrently report nucleic acid encapsulation efficiency, the number of RNA copies per EV, the proportion of EVs containing the target RNA, EV recovery, and functional delivery efficiency.

The loading efficiency of nucleic acids varies among different species. siRNAs and miRNAs are generally more amenable to engineered loading due to their relatively short lengths. In contrast, mRNAs present challenges due to their higher molecular weight, complex structures, and increased susceptibility to degradation, which impose stringent requirements on loading efficiency, RNA integrity, and cytosolic release [135,136]. The main differences between endogenous and exogenous nucleic acid loading strategies are summarized in Table 2.

In addition to nucleic acid loading, the targeting of EVs can be enhanced through peptide conjugation, antibody modification, or aptamer modification. Targeting peptides can be fused to EV membrane proteins such as Lamp2b and CD63. Antibodies or nanobodies can be displayed via membrane-anchoring structures or Fc-binding modules, while aptamers can be attached to the EV surface through lipid anchors [137,138,139,140]. These ligands enhance the binding and uptake of EVs by receptors on target cells [141].

The natural origin of EVs does not inherently guarantee their safety. Factors such as immunogenicity, biodistribution, and toxicity can be influenced by the source of the EVs, the state of the donor cells, the manufacturing and purification processes, the dosage, the route of administration, the targeting ligands, the loading reagents, and any process-related residues [14].

While engineered targeting may enhance local exposure, it cannot completely prevent uptake by the liver, spleen, or mononuclear phagocyte system, and may also introduce additional immune responses or off-target effects [14,142,143]. Special caution is warranted for repeated administrations and for EVs derived from tumor cells, immortalized cells, allogeneic cells, or donor cells in abnormal states. Therefore, safety assessments should be conducted on a product-specific basis, taking into account factors such as tissue accumulation, organ toxicity, immunogenicity, tumor-promoting potential, and the reversibility of adverse effects.

## 5. Application of EV-Associated Nucleic Acids in Respiratory Diseases

**Figure 5:**
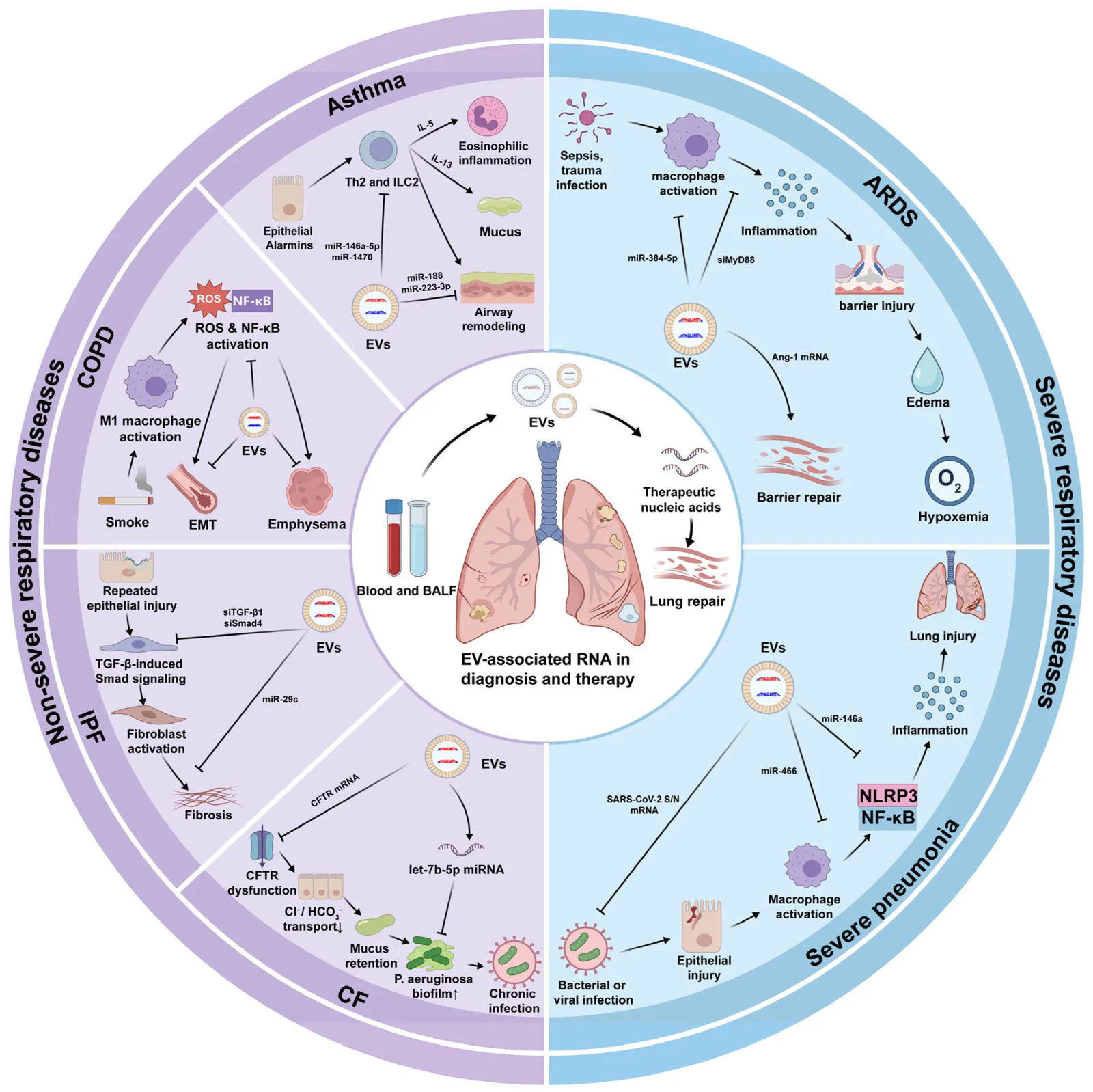
The role of extracellular vesicle-associated nucleic acids in the diagnosis and treatment of respiratory diseases. This figure summarizes the applications of nucleic acids associated with EVs in the diagnosis and treatment of various respiratory diseases. EVs found in body fluids, such as blood, BALF, and mucus, can transport disease-related nucleic acids and reflect pathological changes in lung lesions. Furthermore, natural or engineered EVs can deliver therapeutic nucleic acids to target cells, thereby promoting lung tissue repair and alleviating disease-related damage. The left section of the figure illustrates the application of EV-associated nucleic acids in non-severe or chronic respiratory diseases, including asthma, COPD, IPF, and CF. Conversely, the right section of the figure depicts the application of EV-associated nucleic acids in severe respiratory diseases, including ARDS and severe pneumonia. Abbreviations: ARDS, acute respiratory distress syndrome; BALF, bronchoalveolar lavage fluid; CF, cystic fibrosis; CFTR, cystic fibrosis transmembrane conductance regulator; COPD, chronic obstructive pulmonary disease; DAMP, damage-associated molecular pattern; EMT, epithelial–mesenchymal transition; EV, extracellular vesicle; ILC2, group 2 innate lymphoid cell; IPF, idiopathic pulmonary fibrosis; LPS, lipopolysaccharide; miRNA/miR, microRNA; MyD88, myeloid differentiation primary response 88; NF-κB, nuclear factor kappa B; NLRP3, NLR family pyrin domain containing 3; ROS, reactive oxygen species; siRNA, small interfering RNA; Smad, suppressor of mothers against decapentaplegic; TGF-β, transforming growth factor beta; Th2, T helper type 2 cell.

Respiratory diseases exhibit significant variability in their clinical trajectories, underlying pathological mechanisms, and severity. Consequently, this review delineates non-severe or chronic diseases—such as asthma, COPD, IPF, and CF—from severe conditions, including ARDS and severe pneumonia. The former section concentrates on disease stratification, monitoring, acute exacerbations, and long-term interventions, while the latter emphasizes the assessment of acute injuries, inflammatory regulation, and adjunctive treatments (Figure 5).

### 5.1. Detection and Treatment of Non-Severe or Chronic Respiratory Diseases

Non-severe and chronic respiratory diseases mainly include asthma, COPD, IPF, and CF, among others. Most of these diseases show a long-term persistence or repeated acute exacerbations. Their common pathological basis includes chronic inflammation, airway or alveolar epithelial dysfunction, abnormal ECM deposition, and infection-related injury [144,145]. EVs can transmit pathological signals between airway epithelial cells, immune cells, fibroblasts, and endothelial cells, making them suitable as a platform for linking mechanistic understanding, diagnosis, and therapeutic intervention of chronic lesions [146]. The following will focus on asthma, COPD, IPF, CF, and other diseases, discussing EV-related biomarkers and treatment strategies using natural or engineered EVs.

#### 5.1.1. Asthma

Asthma is a heterogeneous chronic airway inflammatory disease. Its main pathophysiological characteristics include airway hyperresponsiveness, variable airflow limitation, abnormal mucus secretion, and airway remodeling [147]. Recent studies generally suggest that EVs participate in intercellular communication among epithelial, immune, and structural cells in asthma [148,149,150,151], and they can also exist stably in blood and airway samples, gradually becoming an important research focus for asthma diagnosis, subtyping, and therapeutic intervention.

The clinical diagnosis of asthma continues to rely primarily on a history of variable respiratory symptoms and evidence of variable expiratory airflow limitation as demonstrated through pulmonary function testing. While FeNO, blood or sputum eosinophils, and IgE can aid in identifying the T2 inflammatory phenotype, none of these markers can independently establish a diagnosis of asthma [152]. Consequently, EV-associated RNAs currently serve as adjunctive markers for stratifying asthma phenotypes, endotypes, and severity. Vázquez-Mera et al. [153] analyzed serum exosomal miRNAs in 30 healthy controls, 58 patients with mild asthma, and 61 patients with moderate-to-severe asthma. They developed a diagnostic model that incorporates BMI, FEV_1_/FVC ratio, peripheral blood lymphocytes, monocytes, eosinophils, and miR-126-3p to differentiate between patients with mild and moderate-to-severe asthma. This model achieved an AUC of 0.90 (95% CI: 0.83–0.96) for distinguishing moderate-to-severe asthma from mild asthma, with a sensitivity of 0.88 and a specificity of 0.84. However, these findings have not been validated in an independent cohort, and the model also includes pulmonary function and blood-cell indices. Thus, the independent diagnostic contribution of EV-miRNA relative to existing clinical indicators remains uncertain. Additionally, miR-21-5p and miR-126-3p were found to be elevated only in samples collected during autumn from patients with T2-high atopic asthma, but they were decreased in patients with moderate-to-severe asthma characterized by high IL-6 expression. These findings imply that these biomarkers may be influenced by seasonal changes, asthma endotype, obesity, neutrophilic inflammation, and other factors, and their reproducibility requires further validation.

Another study involving 18 patients with moderate-to-severe asthma and 18 healthy controls [154] demonstrated that the AUC values of serum exosomal miR-155 and miR-221 for distinguishing the two groups were 0.91 and 0.76, respectively. However, the corresponding sensitivity, specificity, and optimal cut-off values were not reported, and there was a lack of independent validation. Consequently, it remains uncertain whether these miRNAs are specific to asthma or primarily reflect a shared immune-inflammatory response. Future studies should employ standardized methods for EV isolation and RNA detection. Additionally, it is essential to ascertain whether EV-derived miRNAs enhance the accuracy of asthma phenotyping and severity assessment beyond the current metrics of pulmonary function tests, FeNO, and eosinophil counts. Overall, current evidence regarding serum EV-miRNAs primarily supports their use for exploratory and adjunctive stratification of asthma phenotypes, endotypes, and severity, and is insufficient to advocate for routine clinical application. Furthermore, these EV-associated miRNAs may not only reflect immune-inflammatory states but also directly regulate Th2 inflammation and airway remodeling, thus providing a biological basis for the further development of natural or engineered EV-based treatments for asthma.

In EV treatment studies of asthma, MSC-EVs have received the most attention. MSC-EVs can reshape the airway immune microenvironment and promote regulatory T cell (Treg)-mediated immune regulation, which is associated with increases in immunomodulatory signals such as IL-10 and transforming growth factor β1 (TGF-β1), thereby inhibiting T helper 2 (Th2)-dominated inflammation. This reduces eosinophil infiltration and the release of inflammatory factors such as IL-4 and IL-5 [155]. In this process, miR-146a-5p and miR-1470 contained in MSC-EVs participate in immunomodulation: miR-146a-5p can inhibit Th2 differentiation [156] and limit type 2 innate lymphoid cells (ILC2s)-related inflammatory reactions [157], while miR-1470 contributes to immune homeostasis maintenance. In addition, MSC-EVs can also directly act on tissue cells such as airway smooth muscle cells, and regulate pathways such as STAT3/FGF2/ERK1/2, JARID2/Wnt/β-catenin, and NLR family pyrin domain containing 3 (NLRP3) inflammasome-related pathways via their naturally carried and transferred cargo (including miR-301a-3p, miR-221-3p, miR-188, and miR-223-3p), thereby inhibiting abnormal proliferation, migration, and ECM deposition of airway smooth muscle cells, and reducing chronic airway remodeling such as mucus secretion, collagen deposition, and airway wall thickening [158,159,160,161]. However, at present, most studies have not been analyzed according to asthma endotype stratification. In the future, it will be necessary to further clarify the differences in EV nucleic acid markers among different endotypes.

Compared with natural MSC-EVs, engineered EVs have more explicit advantages in targeted design. In asthma treatment, engineered EVs mainly enhance their therapeutic effect by artificially loading antigens or therapeutic miRNAs. Studies have shown that MSC-derived EVs loaded with antigens can be used for mucosal allergen-specific immunotherapy to induce antigen-specific immune tolerance by reducing IgE and IL-4 levels and improving immunomodulatory signals such as TGF-β [162]. At the same time, human umbilical cord mesenchymal-stem-cell-derived EVs (hUCMSC-EVs) loaded with miR-146a-5p can specifically inhibit the nuclear factor κB (NF-κB)/NLRP3 signaling pathway and reduce the release of inflammatory factors such as IL-6, TNF-α, IL-1β, and IL-18, thus enhancing the inhibitory effect on dust mite-induced airway inflammation [163]. This suggests that engineered EVs can not only enhance delivery efficiency but also provide more targeted interventions for neutrophilic asthma phenotypes and refractory asthma by improving cellular selectivity.

Current evidence suggests that EV nucleic acids may aid in the stratification of asthma phenotypes, endotypes, and severity. In contrast, both natural and engineered MSC-EVs have been shown to modulate immune responses and suppress airway remodeling. However, many studies have been limited by small sample sizes or conducted in animal models, leading to inadequate endotype stratification. Therefore, standardized multicenter validation is necessary, along with the clarification of therapeutic targets and long-term safety considerations.

#### 5.1.2. COPD

COPD is a heterogeneous lung disease whose chronic respiratory symptoms originate from airway or alveolar abnormalities, resulting in persistent and often progressive airflow limitation [164]. The pathological basis of COPD is closely related to chronic inflammation, oxidative stress, small airway remodeling, and alveolar destruction caused by long-term exposure to cigarette smoke and other harmful particles or gases [165]. Compared with asthma, COPD-related EVs are considered to be involved in abnormal signaling between epithelial cells, endothelial cells, mononuclear phagocytes, and neutrophils, and they play a role in the progression of inflammation, small airway fibrosis, and emphysema [166,167].

The diagnosis of COPD necessitates the evaluation of chronic respiratory symptoms alongside a history of exposure to risk factors, including smoking and occupational dust. This is complemented by the confirmation of persistent airflow obstruction via a post-bronchodilator FEV_1_/FVC ratio of less than 0.70. Pulmonary function, symptoms, and exacerbation history are primarily utilized for subsequent disease assessment, while imaging techniques aid in the identification of emphysema and bronchiectasis, as well as in excluding other diseases [164]. Bartel et al. [168] identified 11 differentially expressed miRNAs in BALF EVs from 18 COPD patients and 11 healthy controls. However, the study did not perform ROC analysis, nor did it report sensitivity, specificity, or a validation cohort, thereby positioning it primarily within the realm of candidate biomarker discovery. Notably, this study observed an increase in miR-122-5p in BALF EVs, contrasting with a previous study [169] that reported decreased levels of miR-122-5p in the sputum and plasma of patients experiencing acute exacerbations of COPD. Given that the two studies varied in terms of sample source, study population, and disease stage, their findings cannot be directly compared. Nonetheless, these differences imply that such factors may influence the consistency of EV-miRNA measurements.

In a study of blood EVs, O’Farrell et al. [170] demonstrated that miR-223-3p effectively distinguished AECOPD from stable COPD, achieving an AUC of 0.755. In contrast, the combination of miR-92b-3p, miR-374a-5p, and miR-106b-3p yielded a higher AUC of 0.820. However, the study’s small sample size and lack of independent validation limit its generalizability. Wang et al. [171] reported that serum exosomal miR-1258 distinguished AECOPD from stable COPD with an AUC of 0.851, sensitivity of 74.24%, and specificity of 97.62%. When combined with the NLR, the AUC increased to 0.944, with a sensitivity of 81.82% and specificity of 97.62%. The diagnostic performance of this model was superior to that of independently measured CRP, procalcitonin, white blood cell count, and NLR; however, it also lacked independent multicenter validation. Furthermore, miR-1258 was positively correlated with neutrophil count, white blood cell count, CRP, and NLR, suggesting that it may primarily reflect infection- or neutrophil-associated systemic inflammation rather than COPD-specific alterations. Overall, EV-RNAs are currently more appropriately utilized as adjuncts to conventional indicators for identifying acute exacerbations and stratifying disease severity. Additionally, the involvement of these EV-RNAs in inflammation and lung tissue injury provides a biological basis for future research into EV-based therapeutic strategies.

As in asthma, EV-based therapeutic research in COPD has focused primarily on MSC-derived EVs. These EVs may attenuate small-airway injury and alveolar destruction through anti-inflammatory, antiapoptotic, antioxidant, and tissue-reparative effects. Animal studies show that hUCMSC-EVs can reduce the infiltration of inflammatory cells in the lungs and down-regulate the NF-κB p65/p50-related inflammatory pathway to reduce airway and lung parenchyma inflammation in the smoke exposure COPD model [172], but the underlying mechanism has not been clarified yet. In addition, hUCMSC-EVs can also inhibit lung tissue cell apoptosis by activating the VEGF/VEGFR2-AKT and MEK/ERK pathways in the papain-induced emphysema model, thus reducing emphysema-like damage [173]. In addition to natural MSC-EVs, engineered EVs also show certain potential for alveolar repair. Wnt3a-loaded EVs can improve lung function and reduce alveolar cavity enlargement in elastase-induced emphysema models [174]. In general, whether natural MSC-EVs or engineered EVs, they can intervene in the progression of COPD by regulating inflammatory response, alveolar destruction, and airway remodeling.

In COPD, the significance of EV-associated nucleic acids may reside in their ability to reflect local inflammation, identify acute exacerbations, and assist in severity stratification. Conversely, both natural and engineered EVs have demonstrated potential in suppressing inflammation and facilitating lung tissue repair. Considering the limitations inherent in cross-sectional designs, animal-based evidence, and methodological heterogeneity, it is imperative that stable biomarkers undergo validation in longitudinal cohorts. Furthermore, the effective cargoes, dosages, and routes of administration for therapeutic EVs need to be established.

#### 5.1.3. IPF

IPF is a chronic fatal lung disease characterized by progressive pulmonary interstitial fibrosis. Its core pathological process, unlike in asthma, is not dominated by airway inflammation and immune imbalance [175]. Unlike asthma and COPD, IPF is primarily driven by repetitive injury to the alveolar epithelium, dysregulated repair mechanisms, persistent activation of fibroblasts, and excessive deposition of ECM, rather than by airway inflammation alone [175,176]. Therefore, EV-related research in IPF is more inclined to identify fibrosis signals and evaluate the progression of fibrosis.

The diagnosis of IPF necessitates the exclusion of known etiological factors, such as drug exposure, environmental or occupational hazards, and connective tissue diseases. This diagnostic process is primarily based on HRCT findings, which may be complemented by histopathological analysis and multidisciplinary discussions when warranted [177]. Currently, there is no blood or BALF-based molecular biomarker that can supplant this established diagnostic pathway. However, EV-associated RNAs are potential candidates for aiding in the identification of IPF and its acute exacerbations.

Gan et al. [62] found that plasma exosomal hsa_circ_0044226, hsa_circ_0004099, and hsa_circ_0008898 exhibited AUCs of 0.936, 0.887, and 0.934, respectively, for diagnosing IPF in a cohort of 113 patients with IPF and 76 controls. The combination of these three circRNAs yielded an AUC of 0.991, with a sensitivity of 98.23% and a specificity of 93.42%. Notably, the AUC of hsa_circ_0044226 for distinguishing acute exacerbation of IPF from stable IPF was 0.952, demonstrating a sensitivity of 100% and a specificity of 88.89%. Furthermore, its expression was negatively correlated with pulmonary function parameters, including FVC and FEV_1_. However, it is important to note that this was a single-center study, and its validation cohort included samples utilized in the preliminary screening and training phases. Additionally, the controls predominantly consisted of non-IPF volunteers. Therefore, the findings do not conclusively demonstrate that these circRNAs can differentiate IPF from other fibrotic interstitial lung diseases. A previously reported peripheral blood circRNA, hsa_circ_0058493 [178], was elevated in both IPF and silicosis; however, Gan et al. [62] found no significant difference in plasma exosomal hsa_circ_0058493. These findings suggest that candidate circRNAs may be influenced by various factors, including the analyte being measured and the sample source, indicating that their reproducibility necessitates independent multicenter validation. Overall, while EV-circRNAs cannot currently replace HRCT and multidisciplinary diagnosis, they may complement pulmonary function testing and imaging in the identification of IPF and its acute exacerbations. Additionally, the functional involvement of certain profibrotic circRNAs provides a foundation for further research on EV-based treatments.

In disease treatment, unlike MSC-EVs in asthma and COPD that often play a role through anti-inflammatory, immunomodulatory, and tissue repair effects, EV treatment studies in IPF are more inclined to block pro-fibrotic signals, reduce the persistence of myofibroblasts, and improve the state of senescent or damaged alveolar epithelial cells. Cell-derived EVs, whether natural or loaded with non-specific therapeutic nucleic acids, remain one of the most studied categories. In 2023, Basalova et al. [179] reported that MSC-EVs promote the regression of existing pulmonary fibrosis, which is related to the intercellular transport of miR-29c and miR-129, reducing myofibroblasts and fibroblast activation protein α (FAPα)-positive progenitor cells, and promoting myofibroblast dedifferentiation. In addition to natural MSC-EVs, engineered EVs can further improve the anti-fibrotic effect by loading specific therapeutic nucleic acids or through surface modifications, which has become an important direction for IPF nucleic acid delivery research. In 2024, Qiu et al. [180] developed a milk-derived exosome-siTGF-β1 system for nebulized administration. They achieved pulmonary siRNA delivery in a pulmonary fibrosis model, thereby silencing TGF-β1 expression, inhibiting epithelial–mesenchymal transition (EMT) reducing inflammatory infiltration and ECM deposition, and improving animal survival. In addition, some studies have attempted to adopt a timed delivery strategy [181]. They first delivered engineered exosomes (ExoMMP19) carrying matrix metalloproteinase 19 (MMP19) on their surfaces to weaken the excessive ECM barrier in fibrotic lung tissue and then delivered therapeutic exosomes (ExoTx) with surface D-mannose modification and loaded with siDrp1. Lung fibrosis foci are enriched with CD206^+^ fibrotic macrophages. These cells have high mitochondrial content, high dynamin-related protein 1 (Drp1) expression, and more active mitochondrial fission, which helps maintain a fibrotic state. ExoTx is more likely to enter such fibrotic macrophages in vivo, thereby inhibiting Drp1-induced abnormal mitochondrial fission and reducing pulmonary fibrosis.

In the context of IPF, EV-associated nucleic acids may facilitate both diagnosis and the assessment of disease progression. Conversely, natural or engineered EVs have the potential to deliver antifibrotic nucleic acids, thereby intervening in the fibrotic process. Given that the current evidence primarily stems from single-center studies and animal models, future research should focus on optimizing lung-targeted delivery, determining the appropriate timing for treatment, and evaluating long-term safety.

#### 5.1.4. CF

CF is an autosomal recessive monogenic disease caused by mutations in the cystic fibrosis transmembrane conductance regulator (CFTR) gene. CFTR primarily mediates chloride (Cl^−^) and bicarbonate (HCO_3_^−^) transport across the apical membrane of epithelial cells. Defective function leads to disruption of airway surface liquid homeostasis, increased mucus viscosity, impaired mucociliary clearance, and repeated lung infections [182,183]. Pulmonary manifestations include mucus retention, recurrent bacterial infections, and persistent neutrophilic inflammation [182,183]. Compared with the above diseases, the focus of CF-related EV research is more on reflecting infection activity, neutrophil protease load, abnormal ion transport, and molecular changes following CFTR modulator treatment.

The diagnosis of CF primarily relies on clinical manifestations, sweat chloride testing, CFTR genetic analysis, and other assessments of CFTR function [184]. EV-associated RNAs are currently utilized mainly for the identification of pulmonary exacerbations and monitoring of disease status. Stachowiak et al. [185] demonstrated that a combined model incorporating let-7c, miR-16-5p, miR-25-3p, and miR-146a in serum EVs effectively identified pulmonary exacerbations in children with CF, achieving an AUC of 0.96. The combination of sputum miR-25-3p with FVC or FEV_1_ enhanced the AUC from 0.77 and 0.80 to 0.84 and 0.88, respectively. Furthermore, the AUC reached 0.90 for the model combining serum miR-146a with CRP and 0.88 when serum miR-146a was paired with neutrophilia. However, initial sequencing analyses did not reveal significant differences in sputum or serum miRNAs between pulmonary exacerbation and stable disease. Subsequent validation in a larger sample indicated a significant reduction in serum miR-146a, suggesting that the initial screening and validation findings were not entirely consistent. However, the initial sequencing analysis revealed no significant differences in serum or sputum miRNAs between clinically stable periods and exacerbation phases. Additionally, some candidate signals emerged only during subsequent validation, suggesting a limited robustness of the findings. The study was constrained by a small sample size and did not report sensitivity, specificity, or defined thresholds. Furthermore, it lacked independent external validation and clinically relevant control groups, such as patients with non-CF bronchiectasis or other pulmonary infections. Consequently, it remains unclear whether these miRNAs specifically reflect pulmonary exacerbations in CF or primarily indicate infection and neutrophilic inflammation.

In addition to studies on EV-miRNA, a limited number of original investigations have explored the diagnostic relevance of EV protein cargo in CF. Trappe et al. [186] conducted an initial characterization of serum EVs from patients with CF, identifying differences in EV abundance and protein composition between children and adults. They also observed changes in certain EV proteins before and after treatment with the CFTR modulator Kaftrio, suggesting that serum EVs may reflect age-related disease status and treatment responses. However, this exploratory pilot study did not establish a biomarker model with defined thresholds, diagnostic performance, or external validation. Ben-Meir et al. [187] further analyzed paired sputum samples collected from 21 children with CF before and after treatment for pulmonary exacerbations. Their findings indicated that levels of EV-associated neutrophil elastase and myeloperoxidase changed following treatment, and alterations in EV-associated neutrophil elastase were correlated with improvements in lung function. This suggests that sputum EV cargo may be valuable for dynamically monitoring airway inflammation and treatment responses. Nevertheless, the study had a small sample size, focused on proteins rather than nucleic acids, and demonstrated complex patterns of post-treatment changes in EV proteins, highlighting the need for validation in independent cohorts.

Animal studies have provided limited supporting evidence for the role of EV-associated RNAs in CF. Mao et al. [188] discovered that BALF exosomes from Scnn1b-transgenic mice, which exhibit CF-like mucoinflammatory lung disease, were enriched in proteins linked to macrophage activation, neutrophil responses, and mucoinflammation. This suggests that the cargo of airway EVs can reflect local pathological changes. However, it is important to note that the study focused on the EV proteome, and the Scnn1b-transgenic mouse model primarily simulates CF-like mucus obstruction and inflammation rather than the CFTR mutations themselves. Consequently, these findings do not provide direct evidence for the diagnostic value of EV-associated RNAs in CF. Currently, the diagnostic evidence for EV-associated RNAs in CF lung disease is largely derived from a single small-scale pediatric study and is considerably more limited compared to that available for asthma, COPD, and IPF. At this stage, EV-associated miRNAs should be considered as candidate adjunctive biomarkers that complement lung function and inflammatory indicators. Future multicenter longitudinal studies are essential to validate their potential for diagnosing disease and predicting pulmonary exacerbations.

In terms of treatment, unlike MSC-EVs in the above diseases, which are mainly used for anti-inflammatory, repair, or anti-fibrosis, EV treatment in CF is mainly focused on controlling Pseudomonas aeruginosa-related infection, reducing airway inflammation, and restoring or enhancing CFTR function. Koeppen et al. [189] found that EVs derived from human airway epithelial cells delivered let-7b-5p to Pseudomonas aeruginosa, which reduced the expression of proteins associated with biofilm formation and the β-lactamase AmpC, thereby suppressing biofilm formation and increasing bacterial susceptibility to β-lactam antibiotics. The same group subsequently discovered that EVs also delivered a small RNA fragment derived from an rRNA precursor, which increased the susceptibility of *P. aeruginosa* to ciprofloxacin by downregulating proteins associated with the MexHI–OpmD drug efflux pump [190]. These studies demonstrate that airway epithelial cell-derived EV-associated RNAs can directly modulate bacterial phenotypes and antimicrobial susceptibility. Sarkar et al. [191] further showed in *P. aeruginosa*-infected CF mice that intratracheal administration of EVs derived from normal human bronchial epithelial cells reduced pulmonary bacterial burden, levels of inflammatory mediators, and infection-associated body weight loss, thereby extending the evidence from in vitro mechanistic studies to an animal infection model. However, the EVs used in this animal study contained multiple nucleic acids, proteins, and lipids; therefore, it remains unclear whether their in vivo antimicrobial effects were mediated by let-7b-5p, the rRNA fragment, or other specific cargo. Additionally, the study did not demonstrate sustained efficacy against chronic biofilm infection in CF.

On this basis, engineered EVs or exosomes are mainly used to compensate for CFTR function. Studies conducted by Vituret et al. [192] have demonstrated that EVs, microvesicles, and exosomes can effectively deliver exogenous CFTR glycoprotein and its encoding mRNA, specifically mRNA^GFP-CFTR^, into CF cells. This delivery results in the correction of CFTR chloride channel function; the initial function is primarily attributed to the transferred mature CFTR protein, while the subsequent function arises from the translation of GFP-CFTR from the exogenous mRNA. In addition, Villamizar et al. [193] constructed MSC-derived exosomes delivering the CFTR-targeting zinc finger protein transcription activator CFZF-VPR. This transcriptional activator targets the CFTR promoter, thereby enhancing endogenous CFTR expression in bronchial epithelial cells. However, it remains to be determined whether this strategy can achieve sustained functional correction or improve infection-related outcomes. Currently, the available evidence primarily derives from in vitro and animal studies, and further validation is required for clinical translation.

Research on EVs in CF has evolved from merely monitoring pulmonary exacerbations and the burden of neutrophilic inflammation to the delivery of CFTR protein, mRNA, and transcriptional regulators. However, the existing evidence is predominantly derived from in vitro and animal studies, and the applicability of these findings across various CFTR mutations remains unestablished. Future research should focus on enhancing airway delivery efficiency and durability, expanding genotype coverage, and advancing clinical validation.

### 5.2. Detection and Treatment of Severe Respiratory Diseases

Severe acute respiratory diseases mainly include ARDS and severe infectious pneumonia. Unlike chronic diseases, these conditions usually have an acute onset and rapid progression. The common pathological basis is the destruction of the alveolar-capillary barrier and an uncontrolled inflammatory response caused by infection, sepsis, or other acute injuries. After alveolar macrophages, neutrophils, alveolar epithelial cells, and vascular endothelial cells are activated, they can release large quantities of inflammatory factors, reactive oxygen species (ROS), and proteases, further aggravating alveolar edema, hypoxemia, and diffuse lung damage [194,195]. However, there is still a lack of targeted drugs that can directly promote the repair of the alveolar-capillary barrier and improve prognosis in clinical practice. The following will summarize the diagnosis and treatment progress of severe respiratory diseases from the perspective of EVs and their nucleic acid cargo.

#### 5.2.1. ARDS and Acute Lung Injury (ALI)

ARDS is the most representative acute inflammatory lung injury syndrome among severe respiratory diseases. The main pathological changes are damage to the alveolar epithelium and pulmonary capillary endothelial barrier, leading to protein-rich pulmonary edema, diffuse alveolar injury, surfactant dysfunction, and hypoxemia [194]. In preclinical research, the term ALI is frequently employed to describe experimental models that replicate specific pathological features of clinical ARDS. Models of ALI induced by lipopolysaccharides (LPS), infections, sepsis, and mechanical ventilation offer mechanistic insights and therapeutic evidence pertinent to ARDS, although they do not completely mimic the clinical syndrome. At present, ALI is no longer considered an independent diagnosis separate from ARDS in clinical practice; instead, acute diffuse inflammatory lung injury is uniformly classified as ARDS and further divided into mild, moderate, and severe based on the degree of hypoxemia [196].

The diagnosis of ARDS primarily relies on the identification of an acute predisposing risk factor, the onset or exacerbation of hypoxaemic respiratory failure within one week, bilateral opacities on chest imaging, and abnormal oxygenation, while excluding respiratory failure primarily due to cardiogenic pulmonary oedema or fluid overload [197]. Although CRP and procalcitonin may indicate inflammation or infection, they cannot definitively diagnose ARDS; moreover, EV-associated RNAs remain potential adjunctive biomarkers. Parzibut et al. [198] initially screened plasma exosomal miRNAs in eight ARDS patients and ten healthy controls, followed by validation in an independent cohort of 15 patients and 20 healthy controls. The AUCs for seven miRNAs in distinguishing ARDS from healthy controls ranged from 0.842 to 0.993. However, sensitivity, specificity, and defined thresholds were not reported, and the validation was conducted in a small, single-centre cohort. The initially identified miR-142-5p, miR-223-3p, and miR-486-5p could not be replicated in the validation cohort, indicating limited stability of these candidate biomarkers. Additionally, some of these miRNAs are associated with systemic inflammation, hepatocellular injury, and multiple-organ dysfunction, suggesting that their high AUCs may reflect the inflammatory burden of critical illness rather than ARDS-specific alterations.

In a study examining severe pneumonia-associated ARDS, Sun et al. [61] investigated 38 patients alongside 38 healthy controls. They discovered that the BALF exosomal hsa_circRNA_042882 exhibited an AUC of 0.805, demonstrating a sensitivity of 90% and a specificity of 65%. In contrast, hsa_circRNA_104034 displayed an AUC of 1.000, with a sensitivity of 85% and a specificity of 90%. The corresponding plasma biomarkers showed AUCs of 0.835 and 0.799, respectively. Notably, the study did not perform direct comparisons of these biomarkers with CRP, procalcitonin, oxygenation indices, or imaging-based models, and it focused solely on patients with severe pneumonia-associated ARDS and healthy controls. Following an expansion of the sample size, three additional candidate circRNAs could not be reproduced, suggesting that their reproducibility must be validated in multicenter studies involving clinically relevant comparator populations. This pathological process also indicates potential avenues for clinical intervention.

MSC-derived EVs are among the most extensively studied EV-based interventions for ARDS. They may exert anti-inflammatory, immunomodulatory, anti-infective, and barrier-restorative effects. Bone marrow mesenchymal-stem-cell-derived exosomes (BMSC-Exos) can inhibit NF-κB-mediated inflammatory responses by activating the Nrf2/ARE antioxidant defense pathway, thereby reducing LPS-induced pulmonary epithelial cell damage and ALI [199]. BMSC-Exos can also regulate the damage process of pulmonary epithelial cells and alveolar macrophages through different miRNA cargoes. In pulmonary epithelial cells, BMSC-Exos can deliver miR-182-5p and miR-23a-3p, jointly inhibit IKKβ/NF-κB signal activation, and reduce epithelial damage, EMT, and fibrosis caused by LPS [200]. In addition, miR-384-5p in BMSC-Exos can act on alveolar macrophages to relieve LPS-induced autophagic stress by targeting Beclin-1, reduce macrophage damage and inflammatory response, and thus improve ALI pathological damage [201]. In terms of barrier repair, Angiopoietin-1 (Ang-1) mRNA serves as a notable mRNA cargo associated with EVs. Tang et al. [202] demonstrated that microvesicles derived from MSCs are rich in Ang-1 mRNA. In contrast, microvesicles lacking Ang-1 mRNA were ineffective in restoring pulmonary capillary permeability in LPS-induced ALI models and were also unable to preserve the integrity of pulmonary microvascular endothelial cells under endotoxin stimulation. These findings suggest that EV-associated Ang-1 mRNA plays a critical role in the repair of ARDS by stabilizing the pulmonary microvascular endothelial barrier [202]. Collectively, these findings suggest that MSC-EVs may mitigate epithelial and macrophage injury while facilitating the restoration of the alveolar-capillary barrier through diverse RNA and protein cargoes.

On the basis of natural EV research, engineered EVs currently mainly improve the delivery efficiency of nucleic acid drugs to lung inflammatory cells by enhancing lung deposition, cellular uptake, and target cell recognition. Han et al. [203] loaded siMyD88 into sEVs and delivered them via inhalation, resulting in small EVs being mainly distributed in lung tissue and entering pulmonary macrophages and airway epithelial cells. siMyD88 further downregulates MyD88, an upstream inflammatory signaling molecule, and weakens the overactivation of the NF-κB pathway, thereby alleviating LPS-induced lung damage. On this basis, ligand modification of the EV surface can further improve its ability to recognize specific lung cells. The mannose-modified exosome constructed by Lin et al. [141] can enhance its affinity for alveolar macrophages through mannose-CD206 recognition. After delivering miR-23b-3p, it inhibits the NF-κB pathway, thereby suppressing M1 macrophage activation and reducing the release of pro-inflammatory factors such as IL-1β, IL-6, and TNF-α, leading to decreased pulmonary edema, inflammatory infiltration, and protein exudation.

The clinical value of nucleic acids derived from ARDS-associated EVs primarily lies in their potential to assist in disease identification and assessment of disease progression. In contrast, both natural and engineered EVs may play a role in modulating inflammation and repairing the alveolar–capillary barrier. Given the substantial heterogeneity in the aetiology and progression of ARDS, coupled with the predominance of therapeutic evidence derived from acute lung injury models, future studies should standardize the preparation of EVs, their dosing, routes of administration, and efficacy endpoints.

#### 5.2.2. Severe Pneumonia

Severe pneumonia is an infection-associated condition characterized by significant hypoxemia, acute respiratory failure, septic shock, or the necessity for intensive care and organ support [195]. This section examines severe bacterial and viral pneumonia separately, as the immune responses and mechanisms of tissue injury specific to each pathogen may vary [204,205]. Therefore, this section will discuss the role of EVs in disease detection and treatment intervention around severe bacterial pneumonia and severe viral pneumonia, respectively.

The diagnosis of severe pneumonia relies on the identification of infection-related symptoms and the presence of newly developed pulmonary infiltrates on imaging studies. Microbiological testing is essential for determining the etiology, while hypoxemia and organ dysfunction are critical for assessing disease severity [206,207,208]. Although CRP and procalcitonin can serve as adjunctive indicators, they do not replace the necessity for clinical evaluations and imaging assessments. EV-associated RNAs are currently viewed as complementary indicators of local inflammation and disease progression.

Current evidence suggests that EV-associated nucleic acids in pneumonia may reflect local pulmonary inflammation, systemic infection, and disease severity, as well as pathogen-related signals. Therefore, when evaluating candidate RNAs, it is essential to distinguish whether they are intended to identify local pulmonary inflammation, reflect systemic infection and disease severity, or discriminate specific pathogens. Merely observing differences in expression between groups is insufficient to establish pneumonia or pathogen specificity.

In a study by Sun et al. [209], the comparison was made between 61 mechanically ventilated patients diagnosed with pneumonia and 13 non-pneumonia ICU patients, all of whom were critically ill and had undetermined causative pathogens. The researchers found elevated levels of miR-17-5p and miR-193a-5p in BALF-EVs. The AUC for the two miRNAs in identifying pneumonia was 0.753 and 0.692, respectively, with sensitivities of 59.02% and 50.82%. The combination of these two miRNAs yielded an AUC of 0.748, which did not significantly enhance diagnostic performance. Notably, neither miRNA showed significant differences in plasma EVs or total plasma, indicating that miR-17-5p and miR-193a-5p are more likely to reflect local pulmonary inflammation rather than stable circulating or pathogen-specific signals. Due to the small sample size, lack of stratification by bacterial, viral, or mixed infections, the invasive nature of BALF collection, and the limited sensitivity of the candidate markers, the utility of these miRNAs is primarily restricted to adjunctive identification in critically ill patients.

In comparison to local signals found in BALF, circulating EV-associated RNAs may provide a more accurate reflection of systemic infection and disease severity. Hermann et al. [210] enrolled 30 patients diagnosed with CAP, 65 patients suffering from pneumonia-associated sepsis, and 47 healthy controls, subsequently establishing training and independent validation cohorts. Notably, miR-193a-5p and miR-542-3p were primarily effective in differentiating infected patients from healthy individuals, while miR-1246 exhibited a progressive increase from healthy controls to patients with CAP, and further to those with pneumonia-associated sepsis, indicating a potential correlation with disease severity. However, the inclusion of healthy controls limited the assessment of whether these miRNAs could differentiate pneumonia from other infectious or inflammatory conditions. Furthermore, the study did not develop a clinical model with established thresholds, sensitivity, and specificity. The crude EV fraction obtained through precipitation may have also included non-vesicular miRNAs associated with lipoproteins or Argonaute protein complexes; thus, it remains unverified whether all candidate RNAs originated from EVs. Importantly, although miR-193a-5p was altered in the serum crude EV fraction in this study, it did not show a significant difference in the plasma samples analyzed by Sun et al., suggesting that its reproducibility may be affected by the type of sample used (serum versus plasma), patient characteristics, and EV isolation methods.

A study involving 247 children with viral pneumonia provided more direct evidence for severity stratification [211]. Plasma exosomal DLEU2 was found to be elevated in the severe group, effectively distinguishing severe cases from mild ones, with an AUC of 0.839, a sensitivity of 85.0%, and a specificity of 74.8%. Additionally, elevated DLEU2 levels were associated with lower oxygen saturation, as well as increased levels of CRP and IL-6. However, it is important to note that these findings were based solely on internal fivefold cross-validation. Furthermore, adenovirus, influenza virus, and mixed viral infections were more prevalent in the severe group. Since viral subtype was not included in the multivariable adjustment, the DLEU2 levels may have been influenced by both disease severity and pathogen composition. Moreover, changes related to severity may not necessarily follow a unidirectional pattern. For instance, Meidert et al. [212] reported that miR-3168 was elevated in patients with COVID-19 pneumonia compared to healthy controls but decreased after progression to ARDS. Among 31 sequencing-derived candidate miRNAs examined via RT-qPCR, only 17 exhibited changes in the same direction, and only five ultimately satisfied the predefined differential expression criteria. Furthermore, some data regarding bacterial pneumonia and sepsis were derived from previously established cohorts, which may have been affected by variations in sampling time, experimental batch, and sample matrix. Therefore, while circulating EV-associated RNAs may hold potential for severity stratification, their results should be interpreted in the context of pathogen composition and sampling stage, as a single cross-sectional difference is inadequate for establishing a stable and broadly applicable marker of disease severity.

Nonetheless, these studies lay a foundational basis for future therapeutic research involving the use of EVs to modulate inflammation and mitigate barrier injury in cases of severe pneumonia.

In terms of treatment, the current primary treatment for severe bacterial pneumonia remains timely and appropriate antibacterial therapy, source control of infection, and respiratory and circulatory support. EV-based interventions are being investigated as adjuncts to antimicrobial therapy rather than as replacements. Their proposed effects include enhancing macrophage-mediated bacterial clearance, limiting excessive inflammation, and protecting the alveolar-capillary barrier. Gonzalez et al. [213] found in an *E. coli* pneumonia model that intravenous or nebulized delivery of bone marrow- or umbilical cord-derived MSC-EVs reduced lung damage. EVs remained immunomodulatory and repair-promoting after nebulization, reducing bacterial load and pulmonary edema, and improving blood oxygen levels and lung histopathology scores. In addition, for severe pneumonia caused by drug-resistant bacteria, MSC-EVs cannot directly reverse bacterial resistance, but they can regulate the host immune response and enhance bacterial clearance while reducing excessive inflammation [126]. Shi et al. [126] found in a multidrug-resistant Pseudomonas aeruginosa pneumonia model that adipose-derived MSC-EVs are rich in miR-466, which can be taken up by macrophages. By regulating macrophage responses, they reduce the release of pro-inflammatory factors such as TNF-α and MIP-2, shifting macrophages from a strong pro-inflammatory state to an anti-inflammatory, repair-oriented state, while enhancing their phagocytic and bacterial clearance ability.

EV treatment in severe viral pneumonia mainly reduces lung damage by inhibiting excessive inflammation, improving epithelial-endothelial cell communication, and promoting alveolar barrier repair. The existing evidence mainly comes from COVID-19-related severe pneumonia. In a 2024 study, Anggraeni et al. [214] found that the SARS-CoV-2 spike protein and the H1N1 influenza virus hemagglutinin (HA) protein can jointly induce elevated inflammatory factors in pulmonary epithelial cells, impairing the survival and angiogenesis of pulmonary endothelial cells. In this process, Wharton’s jelly-derived MSC-EVs can deliver miR-146a to pulmonary epithelial cells, inhibit TRAF6 and IRAK1, downregulate the NF-κB pathway, reduce the release of inflammatory factors, and alleviate the adverse effects of epithelial cells on endothelial cell function and immune cell recruitment [214]. However, the study employed an in vitro epithelial-endothelial cell coculture model stimulated with viral proteins, rather than using replication-competent viruses or an animal model of pneumonia. Consequently, the findings presented support only the possibility that miR-146a modulates inflammation induced by viral proteins; they do not provide evidence that it can reduce viral burden, enhance oxygenation, or improve survival in vivo.

EVs can also function as mRNA vaccine delivery platforms for the prevention and control of severe pneumonia associated with respiratory viral infections. Tsai et al. [30] constructed an exosome-based vaccine candidate system loaded with SARS-CoV-2-related antigen mRNAs. In this system, S^W1^ mRNA encodes the wild-type SARS-CoV-2 Spike protein, while LSNME mRNA encodes a multi-antigen construct composed of the nucleocapsid protein and fragments of the spike, membrane, and envelope proteins, which are fused with Lamp1. After loading the aforementioned mRNA into 293F cell-derived exosomes and intramuscularly injecting them into mice for immunization, the system induced antibody responses against the spike and nucleocapsid proteins and activated antigen-specific CD4^+^ and CD8^+^ T cell responses [30]. This study suggests that the EV-mRNA platform, by simultaneously inducing humoral and cellular immunity, may provide a viable delivery strategy for the vaccine-based prevention of severe pneumonia associated with respiratory virus infections.

In cases of severe pneumonia, EV-associated nucleic acids may indicate localized inflammatory and immune alterations. Conversely, MSC-EVs may offer supplementary effects by modulating macrophage function, suppressing excessive inflammation, promoting bacterial clearance, and safeguarding the alveolar-capillary barrier. However, since the existing evidence primarily stems from in vitro and animal studies, and systematic comparisons among pathogen-specific forms of severe pneumonia are insufficient, future research should prioritize the evaluation of the efficacy and safety of combining EVs with standard anti-infective therapies.

Representative human studies of EV-associated RNA biomarkers are summarized in Table 3, while preclinical studies of both natural and engineered EV-based interventions are presented in Table 4. Furthermore, Table 5 offers a comparison of candidate RNAs that have been predominantly reported in individual diseases alongside inflammation-related RNAs identified across multiple respiratory conditions.

## 6. Clinical Studies of EV-Associated Nucleic Acids in Respiratory Diseases

### 6.1. Diagnostic and Biomarker Studies

As of 31 May 2026, there have been relatively few registered studies investigating EV-associated nucleic acid biomarkers in respiratory diseases. NCT05058768 is an observational study designed to conduct multi-omics analyses of EVs isolated from the blood, urine, and bronchoalveolar lavage fluid of patients suffering from ALI or ARDS. The aim is to identify molecular features associated with disease onset and progression. Most studies remain focused on candidate-biomarker discovery. Due to inconsistencies in sample processing, EV isolation, and nucleic acid detection methods, no standardized assay with independent validation and clearly defined thresholds has been established.

### 6.2. Therapeutic Studies

Clinical studies of EV-based treatments for respiratory diseases have primarily focused on ARDS and pulmonary fibrosis. The EXTINGUISH ARDS study (NCT05354141) is a Phase III, multicenter, randomized, double-blind, placebo-controlled trial evaluating the safety and efficacy of intravenous administration of ExoFlo, a bone marrow MSC-EV product, in patients with moderate-to-severe ARDS. The preceding Phase II EXIT-COVID-19 study (NCT04493242) enrolled 102 patients with COVID-19-associated moderate-to-severe ARDS. No treatment-related adverse events were reported; however, the trial did not demonstrate a mortality benefit in the overall population, and the reported benefits were limited to post hoc subgroup analyses [215].

In the context of pulmonary fibrosis, a randomized, single-blind, placebo-controlled Phase I study (ChiCTR2300075466) enrolled 24 patients. Nebulized human umbilical cord MSC-EVs were generally well tolerated, and improvements in FVC, maximal voluntary ventilation, and certain symptom scores were observed in the treatment group. However, the small sample size and heterogeneous disease types necessitate further validation of efficacy [216].

It is noteworthy that all of the aforementioned trials utilized naturally derived MSC-EV multicomponent preparations, whose effects may arise from the combined actions of nucleic acids, proteins, lipids, and other constituents, rather than being attributable to any single nucleic acid cargo. Within the scope of this search, no registered trials were identified in which defined RNAs were actively loaded into engineered EVs and administered to patients with respiratory diseases. Consequently, the delivery of specific nucleic acids using engineered EVs remains predominantly at the preclinical stage.

## 7. Search Strategy and Selection Criteria

We conducted a comprehensive search on PubMed for relevant articles published from the inception of the database through 31 May 2026. The search encompassed three primary concepts: (1) terms related to extracellular vesicles, including “extracellular vesicle,” “exosome,” and “small extracellular vesicle”; (2) terms associated with nucleic acids, including “nucleic acid,” “mRNA,” “circular RNA” or “circRNA,” “siRNA,” and “microRNA” or “miRNA”; and (3) terms pertaining to respiratory diseases, including “respiratory disease,” “lung disease,” “asthma,” “chronic obstructive pulmonary disease,” “idiopathic pulmonary fibrosis,” “cystic fibrosis,” “acute lung injury,” “acute respiratory distress syndrome,” and “pneumonia.” These terms were systematically combined using the Boolean operators AND and OR as appropriate.

In addition, the reference lists of relevant articles and key reviews were manually examined. Original studies and high-quality reviews focusing on the biological mechanisms, diagnostic value, or therapeutic applications of extracellular vesicle-associated nucleic acids in respiratory diseases were included. Recent publications were prioritized; however, seminal studies and authoritative guidelines were incorporated as appropriate. Duplicate publications, conference abstracts, articles lacking accessible full text, and studies unrelated to extracellular vesicle-associated nucleic acids or respiratory diseases were excluded.

## 8. Discussion, Conclusions, and Future Directions

This review systematically summarizes the applications of EV-associated nucleic acids in the diagnosis and treatment of respiratory diseases. The main findings include the following: (1) Body-fluid-derived EV nucleic acids (especially miRNA and circRNA) can reflect inflammation, fibrosis, infection, and other pathological states and have potential for use in disease classification and disease monitoring. (2) Natural EVs (mainly MSC-EVs) exert anti-inflammatory, anti-fibrotic, and barrier repair effects through endogenous nucleic acid cargo. (3) Engineered EVs can be loaded with exogenous nucleic acids to improve delivery efficiency and targeting.

However, progress has been uneven across different nucleic acid classes and respiratory diseases. EV-miRNAs have a relatively broad diagnostic evidence base, particularly in asthma and COPD, where several human studies have reported associations with disease phenotype, severity, pulmonary function, and acute exacerbations. Nevertheless, most candidate markers lack multicenter external validation, standardized detection thresholds, and evidence of incremental value beyond established clinical indicators. Plasma EV-circRNAs in IPF and BALF EV-miRNAs or circRNAs in ARDS and severe pneumonia have shown promising preliminary diagnostic performance, but the evidence is derived mainly from small, single-center studies and remains exploratory. EV-mRNAs have been investigated less frequently as diagnostic biomarkers, whereas siRNAs are primarily artificially designed therapeutic molecules and are generally not used for disease diagnosis. In therapeutic applications, natural MSC-EVs for ARDS and pulmonary fibrosis have progressed from animal studies to early clinical evaluation. However, because natural MSC-EVs contain complex mixtures of nucleic acids, proteins, lipids, and metabolites, their effects cannot yet be attributed to a single RNA species. Engineered EV delivery of siRNAs or miRNAs has defined molecular targets and a relatively clear preclinical rationale in ARDS and pulmonary fibrosis, but it remains largely at the animal experimental stage [142,183,184,202].

Moreover, detecting a candidate RNA in therapeutic EVs does not establish that the observed effect is mediated by that RNA. RNA-depletion experiments, sequence-mutant controls, pathway inhibition, and rescue experiments are needed to demonstrate cargo-specific causality. Longitudinal sampling and predefined early observation windows are also required to determine whether EV biomarkers precede clinical deterioration and to characterize early therapeutic responses.

A significant challenge in manufacturing is the scaling of EV production while ensuring consistent composition and biological activity. Given that the cargo and functionality of EVs are influenced by the state of the donor cells and the conditions of their culture, it is essential that process control encompasses donor cell selection, cell expansion, EV collection, purification, and cargo loading. Consequently, laboratory-scale protocols must be translated into reproducible, validated, and Good Manufacturing Practice (GMP)-compliant manufacturing processes. Quality control should extend beyond merely confirming the presence of EVs; products must be evaluated against predefined criteria for identity, purity, cargo content, potency, and safety. Stability studies are necessary to ascertain whether factors such as storage, freeze–thaw cycles, transportation, or nebulization affect EV integrity, nucleic acid stability, or biological activity. Collectively, these data should define critical quality attributes and release specifications, thereby supporting batch-to-batch consistency and comparability following manufacturing modifications.

In addition to large-scale manufacturing, the clinical approval of exosome-based therapeutic products is hindered by the absence of harmonized regulatory classification and evaluation requirements. In the United States, exosome products intended for disease treatment are generally regulated as drugs and biological products, necessitating that related clinical studies be conducted under an investigational new drug application (IND). To date, no exosome therapeutic product has received approval from the FDA. In the European Union, EV preparations are not uniformly classified as advanced therapy medicinal products (ATMPs). Naturally derived human cell EVs that have not undergone substantial manipulation do not conform to the current definition of an ATMP, while the regulatory classification of engineered EVs must be determined on a case-by-case basis according to their final composition and mechanism of action. This classification uncertainty further impacts the quality, nonclinical, and clinical evidence required for a product. Therefore, developers should engage with regulatory authorities, such as the FDA and EMA, early in the clinical translation process to clarify product classification and the approval pathway as soon as possible.

Future studies may integrate artificial intelligence, multi-omics profiling, and single-EV analysis to identify biomarker combinations that are more stable than individual RNAs. Artificial intelligence may facilitate the integration of EV nucleic acid, protein, lipid, and metabolite data, although the resulting models will require interpretability, external validation, and comparison with conventional clinical models. Single-EV sequencing and multiparametric single-vesicle analysis may reveal low-abundance disease-associated EV subpopulations that are masked in bulk measurements, but their clinical use is currently limited by detection sensitivity, technical variability, and insufficient standardization. Spatial transcriptomics does not directly detect EVs, but its integration with EV nucleic acid profiles may help map pathological tissue niches and generate hypotheses regarding the cellular origins, sites of action, and recipient cells of EVs. In therapeutic applications, EV–liposome or EV–lipid nanoparticle hybrid systems may combine the controllable nucleic-acid-loading and intracellular delivery properties of synthetic carriers with selected biological membrane characteristics of EVs. Nevertheless, these hybrid systems introduce additional manufacturing and regulatory complexity, and their advantages for pulmonary delivery require disease-specific validation.

Overall, EV-associated nucleic acids provide a conceptual link between liquid-biopsy biomarkers and nucleic acid therapeutics, but most applications remain at the discovery or preclinical stage. Diagnostic translation will require standardized assays, longitudinal multicenter cohorts, clinically relevant comparator groups, external validation, and evidence of added value beyond existing clinical indicators. Therapeutic translation will require clearly defined product composition, demonstration of cargo-specific mechanisms, scalable manufacturing, validated potency assays, and systematic safety evaluation. Ultimately, successful development will depend on matching the respiratory disease, nucleic acid class, EV source, engineering strategy, route of administration, quality attributes, and clinical endpoints within a coherent translational framework.

## Figures and Tables

**Table 2:** Comparison of endogenous and exogenous strategies for loading nucleic acids into EV.

Comparison Criterion	Endogenous Loading	Exogenous Loading	References
Basic definition	The target nucleic acid is introduced into or expressed in donor cells before EV formation and release, allowing it to be incorporated into EVs during EV biogenesis.	EVs are first isolated from donor-cell culture supernatants or biological fluids, after which the target nucleic acid is loaded into the isolated EVs using physical or chemical methods.	[128]
Methods	Donor-cell transfection, viral transduction, stable genetic engineering, and RNA-binding protein-mediated active sorting.	Direct incubation, electroporation, sonication, freeze–thaw cycling, membrane extrusion, and chemical transfection.	[128]
Applicable nucleic acid types	miRNAs, siRNAs, mRNAs, and circRNAs; longer or structurally more complex RNAs can also be loaded using specific sorting systems.	Primarily applicable to siRNAs, miRNAs, and other short oligonucleotides; mRNAs can also be loaded but generally require more technically demanding procedures.	[135,136]
Loading efficiency	Generally low to moderate, with substantial variability. Loading efficiency is limited by intracellular nucleic acid expression levels in donor cells, endogenous RNA-sorting mechanisms, and EV secretion capacity.	Apparent loading efficiency is generally higher, although it varies considerably across loading methods, nucleic acid types, and measurement approaches.	[132,135,136]
Main advantages	Loading occurs concurrently with EV biogenesis, generally preserving EV membrane integrity and native biological properties. Nucleic acids can be protected by the membrane as EVs form. This approach also facilitates the simultaneous engineering of targeting membrane proteins or active RNA-sorting elements.	Does not require long-term genetic modification of donor cells; offers greater operational flexibility; enables direct control over the type and amount of nucleic acid added; and is suitable for rapid post-isolation modification of EVs.	[128,135,136]
Main limitations	Depends on donor-cell transfection, expression, and endogenous cargo-sorting capacity. A substantial proportion of the target nucleic acid may remain in donor cells, and the amount loaded is difficult to control precisely. Donor-cell processing is complex and may affect EV yield and composition.	Physical treatments may cause EV membrane damage, aggregation, fusion, or particle loss. Chemical reagents and free nucleic acids may remain as contaminants. Some nucleic acids may merely adsorb to the EV surface rather than being encapsulated within the EV lumen, and an additional purification step is usually required.	[128,132,135,136]
Impact on EV integrity	Generally limited, although donor-cell transfection, viral transduction, or stress-inducing treatments may indirectly alter EV composition.	Generally greater and dependent on operational parameters such as voltage, sonication intensity, the number of freeze–thaw cycles, and extrusion conditions.	[132,135,136]

Abbreviations: circRNA, circular RNA; EV, extracellular vesicle; miRNA, microRNA; mRNA, messenger RNA; RBP, RNA-binding protein; siRNA, small interfering RNA.

**Table 3:** Summary of representative human studies investigating EV–associated RNAs as candidate diagnostic biomarkers in respiratory diseases.

Disease	EV Source	RNA Biomarker(s)	Study Design	Study Population	Expression Changes and Diagnostic Performance	Reference
Asthma	Serum exosomes	miR-21-5p, miR-126-3p, and miR-146a-5p.	Cross-sectional observational study	119 patients with asthma, including 58 with mild asthma and 61 with moderate-to-severe asthma; 30 healthy controls	In patients with T2-high allergic asthma, levels of miR-21-5p and miR-126-3p were found to be elevated. Conversely, in the IL-6-high subgroup of patients suffering from moderate-to-severe asthma, levels of miR-21-5p, miR-126-3p, and miR-146a-5p were significantly reduced. A multivariable model that included miR-126-3p was able to distinguish between mild and moderate-to-severe asthma, yielding an AUC of 0.90 (95% CI, 0.83–0.96), with a sensitivity of 88% and a specificity of 84%.	[153]
	Serum exosomes	miR-155 and miR-221	Case–control study	18 patients with moderate-to-severe or severe asthma; 18 healthy controls	Both miR-155 and miR-221 levels were significantly elevated in the asthma group. The AUC for miR-155 was 0.91, while that for miR-221 was 0.76. Additionally, miR-221 exhibited a positive correlation with FVC (r = 0.47).	[154]
COPD	BALF EVs	miR-122-5p, miR-449c-5p, miR-10a-5p, miR-155-5p, miR-425-5p, miR-320a-3p, miR-378a-3p, miR-7-5p, miR-191-5p, miR-128-3p; miR-101-3p	Cross-sectional observational study	18 patients with moderate COPD; 11 healthy controls	The first 10 miRNAs were elevated in BALF EVs from patients with COPD, whereas miR-101-3p was reduced. AUCs, diagnostic cutoffs, sensitivity, and specificity were not reported.	[168]
	Plasma EVs	miR-34a-5p, miR-92b-3p, miR-223-3p, miR-374a-5p, miR-106b-3p, and miR-374b-5p	Case–control study	20 patients with AECOPD and 20 patients with stable COPD; an additional comparison was performed according to GOLD severity	In AECOPD, miR-34a-5p was reduced, whereas miR-92b-3p, miR-223-3p, miR-374a-5p, and miR-106b-3p were elevated. miR-223-3p distinguished AECOPD from stable COPD with an AUC of 0.755. A combined model comprising miR-92b-3p, miR-374a-5p, and miR-106b-3p yielded an AUC of 0.820. miR-374b-5p distinguished moderate from severe COPD with an AUC of 0.798.	[170]
	Serum EVs	miR-1258	Case–control study	66 patients with AECOPD, 42 patients with stable COPD, and 45 healthy controls	miR-1258 levels were highest in the AECOPD group, intermediate in the stable COPD group, and lowest in healthy controls. For distinguishing AECOPD from stable COPD, the AUC was 0.851, with a sensitivity of 74.2% and a specificity of 97.62%. Combining miR-1258 with NLR increased the AUC to 0.944, the sensitivity to 81.82%, and the specificity to 97.62%. The AUC for distinguishing AECOPD from healthy controls was 0.919.	[171]
IPF	Plasma EVs	hsa_circ_0044226, hsa_circ_0004099, and hsa_circ_0008898	Case–control study	113 patients with IPF and 76 healthy controls; among the patients with IPF, 71 had acute exacerbation and 42 had stable disease	All three circRNAs were elevated in IPF. Their individual AUCs for the diagnosis of IPF were 0.936, 0.887, and 0.934, respectively. The three-circRNA combination yielded an AUC of 0.991, with a sensitivity of 98.23% and a specificity of 93.42%. hsa_circ_0044226 was further elevated during acute exacerbation and distinguished acute exacerbation from stable IPF with an AUC of 0.952, a sensitivity of 100%, and a specificity of 88.89%. It was also inversely correlated with FEV_1_ and FVC.	[62]
CF	Serum, sputum, and exhaled breath condensate EVs	let-7c, miR-16-5p, miR-25-3p, and miR-146a	Paired observational study	30 children with CF; 6 in the discovery phase and 24 in the subsequent validation phase	Serum EV miR-146a was reduced during pulmonary exacerbation. The serum miRNA panel distinguished pulmonary exacerbation from the stable state with an AUC of 0.96. When combined with FEV_1_% predicted or FVC% predicted, sputum miR-25-3p yielded AUCs of 0.88 and 0.84, respectively. When combined with a neutrophil-related parameter or CRP, serum miR-146a yielded AUCs of 0.88 and 0.90, respectively.	[185]
ARDS	Plasma exosomes	miR-130a-3p, miR-221-3p, miR-24-3p, miR-98-3p, let-7d-3p, miR-1273a, and miR-193a-5p	Case–control study	Discovery cohort: 8 patients with ARDS and 10 healthy controls; validation cohort: 15 patients with ARDS and 20 healthy controls	All seven miRNAs were elevated in ARDS. In the order listed, their individual AUCs for distinguishing ARDS from healthy controls were 0.842, 0.843, 0.886, 0.910, 0.943, 0.980, and 0.993.	[198]
Severe pneumonia-associated ARDS	BALF exosomes; plasma	hsa_circRNA_042882 and hsa_circRNA_104034	Case–control study	Discovery phase: 4 patients with ARDS and 4 healthy controls; validation phase: 38 patients with ARDS and 38 healthy controls, including 20 matched pairs with BALF exosome samples	hsa_circRNA_042882 was reduced and hsa_circRNA_104034 was elevated in both BALF exosomes and plasma. In BALF exosomes, their AUCs were 0.805 and 1.000, their sensitivities were 90% and 85%, and their specificities were 65% and 90%, respectively. In plasma, their AUCs were 0.835 and 0.799, their sensitivities were 76.32% and 71.05%, and the specificity was 78.95% for both.	[61]
Pneumonia	BALF EVs	miR-17-5p and miR-193a-5p	Observational study	74 mechanically ventilated ICU patients, including 61 patients with pneumonia and 13 non-pneumonia controls	Both miRNAs were elevated in BALF EVs from patients with pneumonia, whereas no appreciable differences were observed in plasma EVs or total plasma. miR-17-5p had an AUC of 0.753, a sensitivity of 59.02%, and a specificity of 84.62%. miR-193a-5p had an AUC of 0.692, a sensitivity of 50.82%, and a specificity of 100%.	[209]
COVID-19 pneumonia and COVID-19-associated ARDS	Serum EV-enriched preparations	miR-3168, miR-146a-5p, let-7e-5p, and others	Prospective observational study	20 patients with COVID-19 pneumonia, 20 patients with COVID-19-associated ARDS, and 20 healthy controls; sequencing was successful in 15, 15, and 18 participants, respectively; day-14 samples were available from 13 patients with ARDS	Compared with healthy controls, miR-3168 was elevated during COVID-19 pneumonia (log_2_FC = 2.28). Compared with COVID-19 pneumonia, miR-3168 was reduced after progression to ARDS (log_2_FC = −2.13), and let-7e-5p was downregulated.	[212]

Abbreviations: AECOPD, acute exacerbation of chronic obstructive pulmonary disease; ARDS, acute respiratory distress syndrome; AUC, area under the receiver operating characteristic curve; BALF, bronchoalveolar lavage fluid; CF, cystic fibrosis; CI, confidence interval; circRNA, circular RNA; COPD, chronic obstructive pulmonary disease; COVID-19, coronavirus disease 2019; CRP, C-reactive protein; EV, extracellular vesicle; FEV_1_, forced expiratory volume in 1 s; FVC, forced vital capacity; GOLD, Global Initiative for Chronic Obstructive Lung Disease; hsa, Homo sapiens; ICU, intensive care unit; IPF, idiopathic pulmonary fibrosis; log_2_FC, log_2_ fold change; miRNA, microRNA; NLR, neutrophil-to-lymphocyte ratio; T2, type 2 inflammation.

**Table 4:** Summary of representative preclinical studies evaluating EV–based interventions and RNA delivery in respiratory diseases.

Respiratory Disease	EV Source	RNA Cargo	Proposed Mode of Action	Study Design	Main Findings	Reference
Asthma	MSC-derived EVs	Specific RNA cargo not identified	Immunomodulation and suppression of Th2 inflammation	In vitro study and an animal model of allergic asthma	MSC-derived exosomes promoted Treg-associated immunosuppression, enhanced IL-10- and TGF-β1-related signaling, and attenuated Th2 inflammation.	[155]
	hMSC-derived sEVs and miR-146a-5p-engineered EVs	miR-146a-5p	Suppression of allergic airway inflammation	In vitro cell studies and allergen-induced mouse models	Native sEVs delivered miR-146a-5p to limit ILC2-driven type 2 inflammation; engineered EVs further inhibited the IRAK1/TRAF6–NF-κB/NLRP3 axis and reduced inflammatory mediators.	[157,163]
	BMSC-derived or ADMSC-derived EVs	miR-223-3p, miR-221-3p, miR-301a-3p, and miR-188	Suppression of inflammation and airway smooth muscle remodeling	In vitro airway epithelial cell studies and OVA-induced animal models	miR-223-3p suppressed inflammasome activation and pyroptosis. miR-221-3p, miR-301a-3p, and miR-188 inhibited airway smooth muscle cell proliferation, migration, and ECM deposition, thereby reducing mucus secretion, collagen deposition, and airway wall thickening.	[158,159,160,161]
COPD	hUCMSC-derived EVs	Specific RNA cargo not identified; part of a complex EV cargo	Anti-inflammatory, anti-apoptotic, and alveolar-protective effects	Cigarette smoke-induced rat model of COPD and papain-induced rat model of emphysema	hUCMSC-derived EVs reduced inflammatory cell infiltration in the lungs and NF-κB activation. Another study showed that they activated VEGF/VEGFR2-related survival signaling and reduced apoptosis in lung tissue.	[172,173]
IPF	MSC-derived EVs	miR-29c and miR-129	Promotion of the resolution of established pulmonary fibrosis	Fibroblast studies and a BLM-induced animal model of pulmonary fibrosis	Intercellular transfer of miR-29c and miR-129 reduced the numbers of myofibroblasts and FAPα^+^ progenitor cells and promoted myofibroblast dedifferentiation and fibrosis resolution.	[179]
	Milk-derived exosomes	siTGF-β1	Aerosolized siRNA delivery and antifibrotic treatment	In vitro models, including BEAS-2B cells, and a BLM-induced mouse model of pulmonary fibrosis	Nebulized exosomal delivery of siTGF-β1 reduced TGF-β1 expression and Smad signaling, inhibited EMT, inflammatory infiltration, and ECM deposition, and improved animal survival.	[180]
	Sequential ExoMMP19 and D-mannose-modified ExoTx system	siDrp1	Delivery of siDrp1 to profibrotic macrophages to inhibit aberrant mitochondrial fission	In vitro cell studies and a BLM-induced mouse model of pulmonary fibrosis	ExoMMP19 first weakened the barrier formed by excessive ECM deposition; siDrp1-loaded ExoTx subsequently targeted CD206^+^ macrophages, inhibited aberrant mitochondrial fission, and attenuated pulmonary fibrosis.	[181]
CF	EVs from normal human bronchial epithelial cells	let-7b-5p and other small RNAs	Adjunctive anti-infective therapy	In vitro bacterial studies and a CF mouse model of pulmonary infection	EV-mediated delivery of let-7b-5p inhibited Pseudomonas aeruginosa biofilm formation, increased its susceptibility to β-lactam antibiotics, and reduced pulmonary bacterial burden and inflammation in CF mice.	[189]
	EVs from CFTR-positive Calu-3 airway epithelial cells	GFP-CFTR mRNA, together with mature CFTR protein	Delivery of CFTR mRNA to restore CFTR chloride channel function	In vitro studies using human bronchial epithelial cells from patients with CF	Early functional recovery in recipient cells was primarily attributable to the transferred mature CFTR protein, whereas subsequent recovery was associated with the translation of exogenous GFP-CFTR mRNA.	[192]
ARDS	BMSC-derived exosomes	Specific RNA cargo not identified; part of a complex EV cargo	Antioxidant and anti-inflammatory treatment	LPS-stimulated lung epithelial cells and an LPS-induced mouse model of ALI	BMSC-derived exosomes activated Nrf2/ARE antioxidant defenses and inhibited NF-κB signaling, thereby attenuating lung epithelial injury and pathological changes in ALI.	[199]
	BMSC-derived exosomes	miR-182-5p, miR-23a-3p, and miR-384-5p	Suppression of epithelial injury, EMT, and macrophage inflammation	LPS-stimulated lung epithelial cells or alveolar macrophages and LPS-induced animal models	miR-182-5p and miR-23a-3p cooperatively inhibited IKKβ/NF-κB signaling and EMT. miR-384-5p targeted Beclin-1, attenuated autophagic stress and inflammation in macrophages, reduced the increase in pulmonary permeability, and improved animal survival.	[200,201]
	MSC-derived EVs	Ang-1 mRNA	Repair of the alveolar–capillary barrier	LPS-induced mouse model of ALI	EVs containing Ang-1 mRNA reduced pulmonary capillary permeability and protected the endothelial barrier; the corresponding protective effect was markedly attenuated in EVs lacking Ang-1 mRNA.	[202]
	Nebulized engineered sEVs; mannose-modified exosomes	siMyD88; miR-23b-3p	Inhaled small-RNA delivery and macrophage-targeted therapy	In vitro studies and LPS- or sepsis-associated animal models of ALI	Nebulized siMyD88-loaded sEVs were deposited primarily in the lungs and taken up by macrophages and epithelial cells, reducing MyD88/NF-κB activation. Mannose modification enhanced exosome uptake by CD206^+^ macrophages, and miR-23b-3p suppressed M1 polarization, pulmonary edema, and inflammatory injury.	[141,203]
Severe bacterial pneumonia	Bone marrow- or umbilical cord MSC-derived EVs	Specific RNA cargo not identified; part of a complex EV cargo	Adjunctive anti-infective therapy and barrier protection	*E. coli*- or LPS-induced rodent models of lung injury	Both intravenously administered and nebulized MSC-derived EVs reduced bacterial burden and pulmonary edema, improved oxygenation, and ameliorated histopathological lung injury; their immunomodulatory and reparative effects were retained after nebulization.	[213]
Multidrug-resistant Pseudomonas aeruginosa pneumonia	ADMSC-derived EVs	miR-466	Enhancement of bacterial clearance and suppression of excessive inflammation	In vitro macrophage studies and an animal model of multidrug-resistant *P. aeruginosa* pneumonia	Following uptake by macrophages, miR-466-enriched EVs reduced TNF-α and MIP-2, shifted the inflammatory response toward a reparative state, and enhanced phagocytosis and bacterial clearance.	[126]
Severe viral pneumonia	Wharton’s jelly MSC-derived EVs	miR-146a	Suppression of viral protein-induced inflammation and endothelial injury	In vitro coculture model of lung epithelial and endothelial cells	EV-mediated delivery of miR-146a inhibited TRAF6/IRAK1–NF-κB signaling, reduced the release of inflammatory mediators by epithelial cells, and mitigated the resulting adverse effects on endothelial cell survival and angiogenesis.	[214]
Severe pneumonia associated with respiratory viral infection	293F cell-derived exosomes	SW1 mRNA and LSNME multivalent antigen mRNA	Prophylactic mRNA vaccine delivery rather than treatment of established pneumonia	In vitro validation of protein expression and intramuscular immunization in mice	Exosomal delivery of SARS-CoV-2 antigen mRNAs induced antibody responses against the spike and nucleocapsid proteins and activated antigen-specific CD4^+^ and CD8^+^ T cells.	[30]

Abbreviations: ADMSC, adipose-derived mesenchymal stromal cell; ALI, acute lung injury; Ang-1, angiopoietin-1; ARE, antioxidant response element; BEAS-2B, human bronchial epithelial cell line; BLM, bleomycin; BMSC, bone marrow-derived mesenchymal stromal cell; CD206, cluster of differentiation 206; CF, cystic fibrosis; CFTR, cystic fibrosis transmembrane conductance regulator; Drp1, dynamin-related protein 1; ECM, extracellular matrix; EMT, epithelial–mesenchymal transition; EV, extracellular vesicle; ExoMMP19, matrix metalloproteinase-19-displaying pathfinder exosome; ExoTx, D-mannose-modified therapeutic exosome loaded with siDrp1; FAPα, fibroblast activation protein alpha; GFP, green fluorescent protein; hMSC, human mesenchymal stromal cell; hUCMSC, human umbilical cord-derived mesenchymal stromal cell; IKKβ, inhibitor of nuclear factor-κB kinase subunit beta; IL-10, interleukin-10; ILC2, group 2 innate lymphoid cell; IRAK1, interleukin-1 receptor-associated kinase 1; LPS, lipopolysaccharide; LSNME, an mRNA construct encoding the SARS-CoV-2 nucleocapsid protein and selected fragments of the spike, membrane, and envelope proteins; MIP-2, macrophage inflammatory protein 2; MSC, mesenchymal stromal cell; MyD88, myeloid differentiation primary response 88; NF-κB, nuclear factor kappa B; NLRP3, NLR family pyrin domain containing 3; Nrf2, nuclear factor erythroid 2–related factor 2; OVA, ovalbumin; sEV, small extracellular vesicle; siRNA, small interfering RNA; siDrp1, Drp1-targeting siRNA; siMyD88, MyD88-targeting siRNA; siTGF-β1, TGF-β1-targeting siRNA; SW1, mRNA encoding the full-length spike protein of the Wuhan-1 SARS-CoV-2 isolate; TGF-β1, transforming growth factor beta 1; Th2, type 2 helper T cell; TNF-α, tumor necrosis factor alpha; TRAF6, tumor necrosis factor receptor-associated factor 6; Treg, regulatory T cell; VEGF, vascular endothelial growth factor; VEGFR2, vascular endothelial growth factor receptor 2.

**Table 5:** Summary of disease-associated and cross-disease EV-associated RNA candidates reported in respiratory diseases.

Category	EV-Associated RNA	Disease and Sample Type	Major Disease Process Potentially Reflected	References
Candidate RNAs with relative disease specificity	miR-21-5p and miR-126-3p	Asthma; serum EVs	May reflect Th2-associated immune inflammation and differences among asthma endotypes.	[153]
	miR-1258	COPD; serum EVs	May reflect AECOPD-associated neutrophilic and systemic inflammation.	[171]
	hsa_circ_0044226, hsa_circ_0004099, and hsa_circ_0008898	IPF; plasma EVs	May reflect pulmonary fibrosis and disease progression and may participate in TGF-β1-associated fibroblast activation and ECM deposition.	[62]
	let-7c, miR-16/miR-16-5p, and miR-25-3p	CF; EVs from serum, sputum, and exhaled breath condensate	May reflect pulmonary exacerbations, airway inflammation, and changes in lung function in CF.	[185]
	miR-130a-3p, miR-24-3p, miR-98-3p, let-7d-3p, and miR-1273a	ARDS; plasma EVs	May reflect systemic inflammation, immune-cell activation, and processes related to injury repair or fibrosis.	[198]
	hsa_circRNA_042882 and hsa_circRNA_104034	Severe pneumonia-associated ARDS; BALF exosomes and plasma	May reflect hypoxic alveolar injury, inflammatory responses, and HIF-1-related signaling.	[61]
	miR-3168	COVID-19 pneumonia and COVID-19-associated ARDS; serum EV-associated miRNAs	May reflect cytokine imbalance and neutrophil recruitment during progression from COVID-19 pneumonia to ARDS. During the pneumonia stage, miR-3168 was associated with IL-6; after progression to ARDS, its level decreased and was associated with CXCL8.	[212]
Inflammation-related RNAs repeatedly reported across diseases	miR-146a-5p (reported as miR-146a in the CF study)	① Asthma, serum EVs; ② CF, EVs from serum and airway samples; ③ COVID-19 pneumonia and COVID-19-associated ARDS, serum EV-associated miRNAs	May reflect shared immune-inflammatory responses and infection-related acute exacerbations.	[153,185,212]
	miR-221-3p (reported as miR-221 in the asthma study)	① Moderate-to-severe asthma, serum EVs; ② ARDS, plasma EVs; ③ COVID-19-associated ARDS, serum EV-associated miRNAs	May primarily reflect chronic airway inflammation and acute inflammatory lung injury. In COVID-19, it may be associated with ICAM1-related cell adhesion and inflammatory processes.	[154,198,212]
	miR-193a-5p	① ARDS, plasma exosomes; ② Pneumonia, BALF EVs; ③ COVID-19 pneumonia, serum EV-associated miRNAs	May reflect acute pulmonary inflammation, macrophage activation, and lung injury. The COVID-19 study suggested that it may be associated with an IL-10-related immunoregulatory network.	[198,209,212]
	miR-17-5p	① Pneumonia, BALF EVs; ② COVID-19-associated ARDS, serum EV-associated miRNAs	May reflect macrophage-mediated local pulmonary inflammation and changes over the course of ARDS.	[209,212]

Abbreviations: AECOPD, acute exacerbation of chronic obstructive pulmonary disease; ARDS, acute respiratory distress syndrome; BALF, bronchoalveolar lavage fluid; CF, cystic fibrosis; circRNA, circular RNA; COPD, chronic obstructive pulmonary disease; COVID-19, coronavirus disease 2019; CXCL8, C-X-C motif chemokine ligand 8; EBC, exhaled breath condensate; ECM, extracellular matrix; EV, extracellular vesicle; HIF-1, hypoxia-inducible factor 1; ICAM1, intercellular adhesion molecule 1; IL, interleukin; IPF, idiopathic pulmonary fibrosis; miRNA, microRNA; T2, type 2 inflammation; TGF-β1, transforming growth factor beta 1.

## Data Availability

No new data were created or analyzed in this study. Data sharing is not applicable to this article.

## Abbreviations

The following abbreviations are used in this manuscript:

3′ UTR	3′ untranslated region
AECOPD	acute exacerbation of chronic obstructive pulmonary disease
Ago2	Argonaute 2
ALI	acute lung injury
ARDS	acute respiratory distress syndrome
ARE	antioxidant response element
AUC	area under the curve
BALF	bronchoalveolar lavage fluid
BLM	bleomycin
BMSC-Exos	bone marrow mesenchymal-stem-cell-derived exosomes
CF	cystic fibrosis
CFTR	cystic fibrosis transmembrane conductance regulator
circRNA	circular RNA
COPD	chronic obstructive pulmonary disease
CRP	C-reactive protein
DALYs	disability-adjusted life years
ECM	extracellular matrix
EMT	epithelial–mesenchymal transition
EV	extracellular vesicle
FAPα	fibroblast activation protein α
FEV1	forced expiratory volume in 1 s
FLOT1	flotillin-1
FVC	forced vital capacity
hUCMSC-EVs	human umbilical cord mesenchymal-stem-cell-derived extracellular vesicles
ICU	intensive care unit
ILC2s	group 2 innate lymphoid cells
ILVs	intraluminal vesicles
IPA	Ingenuity Pathway Analysis
IPF	idiopathic pulmonary fibrosis
IRES	internal ribosome entry site
LNPs	lipid nanoparticles
LPS	lipopolysaccharide
MPO	myeloperoxidase
mRNA	messenger RNA
MSC-EVs	mesenchymal-stem-cell-derived extracellular vesicles
MVBs	multivesicular bodies
NE	neutrophil elastase
NF-κB	nuclear factor κB
NLR	neutrophil-to-lymphocyte ratio
NLRP3	NLR family pyrin domain-containing 3
PEG	polyethylene glycol
ppFEV1	percent predicted forced expiratory volume in 1 s
RBPs	RNA-binding proteins
RISC	RNA-induced silencing complex
RNAi	RNA interference
ROS	reactive oxygen species
SIRPα	signal regulatory protein α
T2	type 2
TBC1D2B	TBC1 domain family member 2B
TGF-β1	transforming growth factor β1
Th2	T helper 2
Tregs	regulatory T cells
